# Tumor-secreted exosomal miR-141 activates tumor-stroma interactions and controls premetastatic niche formation in ovarian cancer metastasis

**DOI:** 10.1186/s12943-022-01703-9

**Published:** 2023-01-09

**Authors:** Yulan Mo, Leanne L. Leung, Celia S. L. Mak, Xueyu Wang, Wai-Sun Chan, Lynn M. N. Hui, Hermit W. M. Tang, Michelle K. Y. Siu, Rakesh Sharma, Dakang Xu, Stephen K. W. Tsui, Hextan Y. S. Ngan, Mingo M. H. Yung, Karen K. L. Chan, David W. Chan

**Affiliations:** 1 Department of Obstetrics & Gynaecology, LKS Faculty of Medicine, The University of Hong Kong, Hong Kong, SAR China; 2 Centre for PanorOmic Sciences Proteomics and Metabolomics Core, Li Ka Shing Faculty of Medicine, The University of Hong Kong, Hong Kong, SAR China; 3 Faculty of Medical Laboratory Science, Ruijin Hospital, School of Medicine, Shanghai Jiao Tong University, Shanghai, 200030 China; 4 School of Biomedical Sciences, The Chinese University of Hong Kong, SAR Hong Kong, China; 5 School of Medicine, The Chinese University of Hong Kong-Shenzhen, Shenzhen, 518172 China

**Keywords:** miR-141, Hippo/YAP1/pathway, Ovarian cancer, Tumor-stroma interactions, Peritoneal metastases, cancer-associated fibroblasts

## Abstract

**Background:**

Metastatic colonization is one of the critical steps in tumor metastasis. A pre-metastatic niche is required for metastatic colonization and is determined by tumor-stroma interactions, yet the mechanistic underpinnings remain incompletely understood.

**Methods:**

PCR-based miRNome profiling, qPCR, immunofluorescent analyses evaluated the expression of exosomal miR-141 and cell-to-cell communication. LC-MS/MS proteomic profiling and Dual-Luciferase analyses identified YAP1 as the direct target of miR-141. Human cytokine profiling, ChIP, luciferase reporter assays, and subcellular fractionation analyses confirmed YAP1 in modulating GROα production. A series of in vitro tumorigenic assays, an ex vivo model and Yap1 stromal conditional knockout (cKO) mouse model demonstrated the roles of miR-141/YAP1/GROα/CXCR1/2 signaling cascade. RNAi, CRISPR/Cas9 and CRISPRi systems were used for gene silencing. Blood sera, OvCa tumor tissue samples, and tissue array were included for clinical correlations.

**Results:**

Hsa-miR-141-3p (miR-141), an exosomal miRNA, is highly secreted by ovarian cancer cells and reprograms stromal fibroblasts into proinflammatory cancer-associated fibroblasts (CAFs), facilitating metastatic colonization. A mechanistic study showed that miR-141 targeted YAP1, a critical effector of the Hippo pathway, reducing the nuclear YAP1/TAZ ratio and enhancing GROα production from stromal fibroblasts. Stromal-specific knockout (cKO) of Yap1 in murine models shaped the GROα-enriched microenvironment, facilitating in vivo tumor colonization, but this effect was reversed after Cxcr1/2 depletion in OvCa cells. The YAP1/GROα correlation was demonstrated in clinical samples, highlighting the clinical relevance of this research and providing a potential therapeutic intervention for impeding premetastatic niche formation and metastatic progression of ovarian cancers.

**Conclusions:**

This study uncovers miR-141 as an OvCa-derived exosomal microRNA mediating the tumor-stroma interactions and the formation of tumor-promoting stromal niche through activating YAP1/GROα/CXCRs signaling cascade, providing new insight into therapy for OvCa patients with peritoneal metastases.

**Supplementary Information:**

The online version contains supplementary material available at 10.1186/s12943-022-01703-9.

## Background

Ovarian cancer (OvCa) is one of the deadliest gynecologic malignancies in females [1]. Like other solid tumors, cancer metastasis accounts for the great majority of cancer-associated deaths in the patients with OvCa [2]. Transcoelomic or peritoneal metastasis is the most preferential route of the spread of OvCa cells detached from the primary ovaries into the peritoneal cavity [3]. Although most advanced OvCa patients underwent optimal debulking and neoadjuvant could achieve good responses initially, the undetectable chemoresistant or residual tumor cells escaping from standard therapies usually develop micrometastasis causing a high rate of lethal recurrent disease [4, 5]. Thus, OvCa patients with peritoneal metastasis display a higher recurrence rate and are resistant to conventional platinum-based chemotherapy leading to an abysmal prognosis [6, 7]. Metastatic colonization is the crucial step of cancer metastasis, allowing OvCa cells to homing and metastatic colonization of secondary sites in visceral organs and tissues in the peritoneal cavity [8, 9]. Hence, the targeted repression of this critical step could prevent OvCa intraperitoneal micrometastasis and metastatic progression in the peritoneal cavity. Mounting evidence has suggested that cancer cells disseminate preferentially to some specific organs or tissues for metastatic spreading, while the underlying mechanism remains poorly understood [10–12]. Paget’s “seed and soil” metaphor has recently been regarded as a significant contribution to the pathogenesis of peritoneal metastasis of OvCa, especially the step of metastatic colonization for the distal organ-specific and continued uncontrolled tumor growth [13, 14]. Accordingly, metastatic cancer cells disseminate to the secondary sites with a favorable tumor microenvironment (TME) provided by the cellular elements of reactive non-stroma and stroma cells [15, 16]. The stroma, such as cancer-associated fibroblasts (CAFs), act not only as a cancer-accepting tissue but also are an active driver for promoting tumor proliferation, neovascularization, invasion, and immunomodulating antitumor immunity [17, 18]. The reciprocal interactions between the tumor and the stroma establish a local microenvironment that facilitates tumor progression [19]. Nevertheless, the molecular mechanisms implicated in such tumor-stromal interactions in stimulating metastatic progression stay ill-defined.

Extracellular vesicles (EVs), including micro-vesicles, exosomes, and large oncosomes released from malignant cells, have been hypothesized as novel mechanisms of intercellular communication [20]. Recent evidence has revealed that exosomes containing secreted miRNAs act as critical components of the secretome that drive cell-to-cell communication in promoting metastatic progression [21, 22]. Quiescent fibroblasts adjacent to malignant lesions are transformed into CAFs that promotes metastatic progression by multi-processes like pre-metastatic niche activation, mesenchymal-to-epithelial transition (MET) reversion, metastatic colonization, and angiogenesis [21–23]. Although evidence has suggested that dysregulated miRNAs and exosomal miRNAs can mediate functional impacts on CAFs [24], there is not enough evidence showing how tumor-secreted miRNAs remodel normal fibroblasts to be CAF-like phenotype involved in the formation of the pre-metastatic niche.

Here, we report that a cancer-secreted miRNA, miR-141, reprograms stromal cells to a tumor-promoting stromal niche via the miR-141/YAP1/GROα signaling cascade. In contrast, the suppression of CXCR1/2 receptors on OvCa cells slowed tumor progression and dissemination supported by the above cascade, revealing a potential therapeutic approach for preventing OvCa metastasis.

## Materials and methods

### Cell culture

The human embryonic kidney cell line (HEK293), skin fibroblasts (BJ), adipocytes (3 T3-L1), T lymphoblasts (Human Jurkat T-cells; source: male T lymphoblast), endothelial cell line (HUEVC; source: the endothelium of veins from the umbilical cord), human clear cell subtype ovarian cancer cell line ES-2, and three stromal cell lines (WPMY-1; source: male prostate, WI-38; source: female lung, and T HESC; source: female endometrium), were purchased from American Type Culture Collection (ATCC, Manassas, Virginia, USA). A human ovarian clear cell adenocarcinoma cell line, OVMANA, and a high-grade ovarian serous adenocarcinoma (HGSOC) cell line OVKATE, were obtained from the Japanese Collection of Research Bioresources (JCRB) Cell Bank (Tokyo, Japan). One human ovarian cancer adenocarcinoma cell line, CAOV3 [25], was obtained from Dr. Simon Chu, Hudson Institute of Medical Research, Melbourne, Australia. A HGSOC cell line, OVCA433, and three human ovarian cancer adenocarcinoma cell lines, COV413, SKOV3, and A2780cp [25] were obtained from Prof. Benjamin Tsang, University of Ottawa, Canada. A murine high-grade serous subtype cancer cell line, ID8, was obtained from Dr. Katherine F. Roby, The University of Kansas, USA. All cell lines were incubated at 37 °C in a humidified incubator containing 5% CO_2_. WPMY-1, OVCA433, HEK293, HEK293FT, ES-2, SKOV3, ID8, BJ, CAOV3, 3 T3-L1, COV413, were cultured in Dulbecco’s modified Eagle medium (DMEM). A2780cp, OVMANA, OVKATE, and Jurkat T cells were cultured in RPMI 1640 Medium (Gibco). WI-38 cells were cultured in minimum essential medium (MEM) (Gibco). The T HESC cell line and primary human endometrial stromal cells were cultured in Dulbecco’s Modified Eagle Medium: Nutrient Mixture F-12 (DMEM/F12) (Gibco) with 1 mM sodium pyruvate. Mouse primary stromal cells were cultured in RPMI-1640 Medium (Invitrogen) with 50 μM 2-mercaptoethanol, 100 μM asparagine and 2 mM glutamine. All full media were supplemented with 10% fetal bovine serum (FBS) (Gibco) and 1% penicillin-streptomycin (P/S) (Invitrogen).

### Mice

*FSP1-Cre* mice (BALB/c-Tg(S100a4-cre)1Egn/YunkJ, Stock No. 012641) and Yap1^flox^ mice (Yap1^tm1.1Dupa^/J, Stock No. 027929) were obtained from Jackson Laboratories. To generate Yap1 conditional knockout (cKO) in stromal cells, FSP1-Cre mice were crossed with Yap1^flox^ mice to generate a stromal fibroblast-specific knockout of Yap1 in C57BL/6 mice. Yap1^+/+^, Yap1^+/-^, and Yap1^-/-^ mice were generated in the Center for Comparative Medicine Research, The University of Hong Kong. Mice were housed according to the guidelines of the Center for Comparative Medicine Research of The University of Hong Kong. Genomic DNA was extracted from the ear or tail biopsies of the mice for genotyping as previously described [26]. Females aged 5-20 weeks were used throughout this study. All animal experiments were approved by the Committee on the Use of Live Animals in Teaching and Research (CULATR) of The University of Hong Kong (CULATR 4587-18), according to Hong Kong government guidelines.

### Human specimens

Human tissues and body fluids were collected during a surgical operation at the Queen Mary Hospital, operated by the Department of Obstetrics and Gynecology, The University of Hong Kong. All use of clinical samples was approved with the study code numbers UW 11-298 and UW 20-256 by the Institutional Review Board (IRB) from The University of Hong Kong/Hospital Authority Hong Kong West Cluster (HKU/HA HKW IRB).

### PCR-based miRNome profiling

Blood samples were collected from 8 ovarian cancer patients (mixed tumor subtypes, tumor grades =1-3 and tumor stages = 1-4) and 8 healthy female donors. After centrifugation, equal volumes of serum were separated, and total RNA was extracted using a miRNeasy Serum/Plasma Advanced Kit (Cat No. ID: 217204, Qiagen) according to the manufacturer’s instructions. The RNA concentration was measured using a NanoDrop 2000c Spectrophotometer (Thermo Fisher Scientific, USA). Reverse transcription of miRNA was performed by utilizing the miRCURY LNA RT Kit (Cat No./ID: 339340, Qiagen). cDNA samples were used for miRCURY LNA miRNA miRNome PCR Panels (Human Panels and Cancer Focus Panel), and quantitative PCR was performed on a Roche LightCycler 480. The initial data analysis to obtain raw Cq values uses the 2nd derivative method. Data using the 2^-ΔΔCT^ method are presented as relative gene expression and normalized to U6snRNA, SNORD38B, and SNORD49A according to the manufacturer’s protocol. The results were analyzed by the GeneGlobe Data Analysis Center (Qiagen).

### Preparation of the conditioned medium

Completed medium (DMEM, DMEM/F12, MEM, F12K) with 10% FBS was used to maintain the cells. The conditioned medium (CM) was harvested after 3 days. To remove the cell debris and microvesicles, the CM was centrifuged at 1000 rpm for 5 min and filtered using a 220 nm filter. The cleaned conditioned medium was stored at − 80 °C for further use.

To prepare the omentum-conditioned medium (OCM), freshly resected normal omentum or cancerous omentum tissue from Queen Mary Hospital, University of Hong Kong, was washed with PBS once and then cut into small pieces. The minced omentum was mixed with plain medium and incubated at 37 °C with 5% CO_2_ for 24 h. Then, the omentum was removed from the medium using a cell strainer. To remove the sediments, the OCM was centrifuged at 1500 x g for 5 min three times and filtered through a 0.7 μM column. The OCMs were stored at 4 °C for use within 1 month.

### Isolation of exosomes from cell cultures and blood serum

Exosome isolation was performed using differential ultracentrifugation modified from the previous protocol [27]. Briefly, a total of 70 mL culture medium or serum were firstly centrifuged at 300 x g at 4 °C for 10 min to remove intact cells and macroscopic debris, followed by removing other cell debris 2000 x g centrifugation at 4 °C for 20 min. The supernatant was centrifuged at 10000 x g at 4 °C for 30 min, and ultracentrifuged at 100000 x g at 4 °C for 80 min using ultracentrifuge tubes (Hitachi Koki). The pellet remained inside the ultracentrifugation tubes was resuspended with 1 mL PBS and had another ultracentrifugation at 100000 x g at 4 °C for 1 h to wash the exosomes. The exosomes that remained inside the tube were thoroughly resuspended and moved to a 1.5 mL microcentrifuge tube for exosomal RNA extraction.

### RNA extraction and quantitative real-time polymerase chain reaction (qPCR)

The exosomal RNA samples derived from the isolated exosomes were extracted using the miRNeasy Mini Kit (QIAGEN) and analyzed by qPCR according to our previous report [28, 29]. Briefly, miRNAs in total RNA were reverse transcribed to cDNA using the miRCURYLNA™ cDNA Synthesis Kit (Exiqon, Demark) following the manufacturer’s protocol. Briefly, every sample contained 4 μL of 5 X RT reaction buffer and 1 μL enzyme mix, as well as 30 ng of total RNA with DEPC-treated water, filled up to 20 μL total. The mixture was heated at 42 °C for 1 hour, followed by 95 °C for 10 minutes to denature the strand and 4 °C to snap-cool. The synthesized cDNA was cooled to − 20 °C for the study. qPCR targeting mature miR-141 was performed using a miRCURY LNATM SYBR® Green PCR Kit on an ABI7500 System for Real-Time PCR (Applied Systems). The PCR primers miR-141-3p and U6 were purchased from Exiqon (Vedbaek, Denmark). Each 10 μL PCR mixture contained 5 μL SYBR Green Master Mix, 1 μL hsa-miR-141 (Exiqon#204504), and 4 μL diluted cDNA product (1:5). qPCR was performed by heating for 5 minutes at 95 °C, with 40 cycles of a time interval of 10 seconds at 95 °C and an annealing time interval of 1 min set to 60 °C. Results were normalized to U6 snRNA (Exiqon #203907) to measure the relative miR-141 expression.

The total RNA of the cell pellet was extracted using TRIzol RNA extraction reagent and reverse transcribed using SuperScript™ VILO™ MasterMix (Life Technology, CO). Real-time PCR was performed using TaqMan probes provided by TaqMan Gene Expression Assays from Applied Biosystems. Target primer human YAP1 (Assay ID: Hs00902712_g1), target primer human GROα, (Assay ID: Hs00236937_m1), and target primer murine GROα (Assay ID: Mm04207460_m1) were detected using an ABI ABI7500 System of Real-Time PCR (Applied Systems, USA) according to the manufacturer’s instructions. Each 10 μL reaction contained 4 μL of cDNA, 5.5 μL of 2X TaqMan Universal PCR Master Mix (Applied Biosystems Carlsbad), and 0.5 μL of target primer. qPCR was performed by heating for 5 minutes at 95 °C, with 40 cycles of a time interval of 15 seconds at 95 °C and an annealing time interval of 1 min set to 60 °C. Relative expression from the target gene was determined using human GAPDH (Assay ID: Hs02786624_g1), murine GAPDH (Assay ID: Hs99999905_m1), and 18S rRNA (REF:4318839) for normalization. The measurement of the relative expression was calculated using the 2^-ΔΔCt^ method.

### The human XL cytokine array

The conditioned medium (CM) was collected from WPMY-1 cells that overexpressed miR-141, and its scrambled control was collected and filtered in advance. A total of 500 μL of conditioned medium supernatant was applied to the Human XL cytokine array (#894660) using the R&D System, and this assay strictly followed the manufacturer’s protocol.

### Proteomic profiling

The cell pellets from WPMY-1 cells that stably expressed miR-141 and the respective scrambled control were collected, and 1*10^6^ cells were lysed with 200 μL of lysis buffer containing 2 μL of protease and phosphatase inhibitor cocktail. The cell lysates were immediately snap frozen with liquid nitrogen and analyzed by LC-MS/MS (PM CORE, The University of Hong Kong).

### Plasmids

TargetScan 6.0 (http://www.targetscan.org/) was used to identify the potential target of miR141. The plasmids pmir-YAP1-WT and pmir-YAP1-MT were made by cloning annealed synthesized oligos (Supplementary Table S1) into the pmirGLO Dual-Luciferase miRNA Target Expression Vector (Promega, Madison, WI, USA) using SacI and EcoRI. pGL3-GROα(N ~ III) was made by cloning − 1937 to − 288 bp, − 1164 to − 288 bp, − 1119 to − 288 bp, and − 722 to − 288 bp of the GROα promoter into the pGL3-basic vector (Supplementary Table S1) (Promega Corporation, Madison, WI, USA) using NheI and HindIII. YAP1-PX459 or TAZ-PX459 plasmids were made by cloning annealed YAP1 or TAZ CRISPR/Cas9 sgRNA oligos into pSpCas(BB)-2A-Puro(PX459) V2.0 (Supplementary Table S1) (Addgene, Watertown, MA, USA) using BbsI. Cxcr1-Cxcr2-pX330K plasmids were made by cloning Cxcr1 and Cxcr2 CRISPRi sgRNA oligos into pGEP179_pX330K (Addgene) using BbsI (Supplementary Table S1). The sgRNA oligos were designed using the online software E-CRISP Design (http://www.e-crisp.org). All cloned plasmids were amplified and extracted using a QIAGEN Plasmid Midi Kit (QIAGEN) and then sent for sequencing analysis.

### Chromatin immunoprecipitation (ChIP) and the dual-luciferase assays

The transcriptional binding sites of TEAD1 in GROα promoters were scanned using the UCSC genome browser and Ensembl database based on the TEAD1 motif and predicted by MEME Suite (Supplementary Table S2). Three TEAD1 binding sites in the promoter region of GROα (− 1933 to − 1920, − 1140 to − 1127, and − 1012 to − 999) were found spanning from − 2 kb to + 200 bp. A SimpleChIP® Plus Enzymatic Chromatin IP Kit (Cell Signaling Technology, Massachusetts, USA) was used to examine the interaction between TEAD1 and the predicted binding sites on the GROα promoters in T HESCs cells overexpressing TEAD1. The immunoprecipitated chromatin was analyzed by qPCR using specific primers targeting the CXCL1 binding elements (CBEs) on the GROα gene promoters, and the results are illustrated (Supplementary Table S1).

For the dual-luciferase assay, WPMY-1 cells were seeded into 24-well plates and cotransfected with 100 ng pmir-YAP1-WT or pmir-YAP-MT plasmids together with 900 ng pmR-141 plasmid, and pmR-ZsGreen1 empty vector was used as a control. The transfection reagent was Lipofectamine™ 3000 (Invitrogen). To detect the dominant binding site of the YAP1/TAZ/TEAD1 transcriptional complex, WPMY-1 cells were seeded into 24-well plates and cotransfected with 200 ng pGL-GROα(N ~ III), 1 μg pRK5-Myc-TEAD1 and (0, 1, 2 μg) 3x Flag pCMV5-Topo-TAZ (S89A) in a dose-dependent manner. The cell lysates were then transferred to a 96-well plate after 48 h. A Dual-Luciferase Assay Kit (Promega) was used to detect the luciferase activity in each well using a Fluorescence Spectrophotometer F-4500 (Promega). Renilla luciferase activity was used as the reference for normalization of transfection efficiency.

### Stable cell line construction

HEK293FT cells in 6-well plates were cotransfected with the pHR-UCOE-SFFV-dCas9-mCherry-ZIM3-KRAB and pPACKH1™ Lentivector Packaging Kit (SBI System Biosciences) using the ViraDuctin™ Lentivirus Transduction Kit (Cat# LV500A-1) to generate lentiviral particles expressing dCas9 fused to mCherry and the KRAB domain of ZIM3. The next day, the medium was exchanged for an antibiotic-free medium with 10% FBS. After incubating for 48 h, the virus particle-containing medium was harvested. Then, the medium was mixed with ViraDuction™ Lentivirus Transduction Reagent A and ViraDuction™ Lentivirus Transduction Reagent B, and subsequently transferred to the 50-70% confluent ID8 GFP/Luc cells in a 6-well plate and transduced overnight. The cells were washed twice with ViraDuction™ Lentivirus Transduction Reagent C diluted with a complete culture medium and then fed with a fresh medium. Forty-eight to seventy-two hours after transduction, 1.25 mg/ml puromycin (Invitrogen) selection was performed for 72 h. After selection, ID8 GFP/Luc cells were cotransfected with Cxcr1 and Cxcr2 pGEP179_pX330K plasmids using Lipofectamine™ 3000 for 48 h to construct ID GFP/Luc Cxcr1 and Cxcr2 CRISPRi stable cell lines. For the YAP1 shRNA stable knockdown cell line, WPMY-1 cells were transduced with medium containing 5 μg/mL Polybrene® as well as 10 μL/mL shYAP1 lentiviral particles and scrambled control for 48 h and then selected as above. For the YAP1 and TAZ CRISPR/Cas9 knockout cell lines, WPMY-1 cells were transfected with YAP1-PX459 or TAZ-PX459 plasmids and scrambled plasmid for 48 h and then selected as described above. Single colonies were picked and spread.

For stable knockdown of nSMAse2 and YAP1 in CAOV3, OVCA433, and SKOV3 cells, lentiviral particle transfection was performed as mentioned above using 10 μL/mL shnSMAse2 or scrambled control. Stably transfected cells were maintained with full medium containing puromycin (10 μL/mL). Western blotting was performed to verify the knockout efficiency of shnSMAse2 and shYAP1.

### Cell transient transfection

The cells were seeded in 6-well plates at 70% confluence before transfection. Plasmid transfection was performed using Lipofectamine™ 3000 according to the manufacturer’s protocol.

### Protein extraction and immunoblotting

Cells were collected in trypsin-EDTA and centrifuged at 500x g for 5 min. The cells were rinsed with PBS once and centrifuged at 500 x g for 2-3 min. The supernatant was removed to collect the cell pellets, and they were lysed on ice for 30 min using 1x lysis buffer with 1% protease inhibitor. The denatured protein mixtures with blue loading dye and 30x DTT were loaded onto SDS-PAGE gels for electrophoresis and then blotted onto an Immobilon®-FL PVDF membrane (Millipore). After blocking with 5% skim milk, the membrane was incubated with the corresponding primary antibodies overnight, followed by incubation with secondary IRDye® 680 RD IgG goat anti-mouse or IRDye® 800CW IgG goat anti-rabbit antibodies for 1 h. The membranes were scanned for a fluorescent signal using an Odyssey®CLx imaging System (Li-Cor Bioscience).

### Immunofluorescent staining

PKH67 (Sigma–Aldrich, MO) was used to stain OVCA433 extracted exosomes. The staining process for exosomes followed the manufacturers’ protocol. In brief, T HESCs and WI-38 cells were incubated with 106 PKH67-stained exosomes for 1 day. Then, T HESCs and WI-38 cells were seeded on glass covers and washed twice with cold PBS for 5 minutes. VECTASHIELD™ Mounting Medium with DAPI (Vector Laboratories) was used to stain and fix the slides.

For staining for target proteins, WPMY-1 cells with control, miR-141 overexpression, and knockdown YAP1 or primary stromal fibroblasts of Yap1^−/−^ cKO mice were seeded on chamber slides at 30% confluence. The slide was first washed with PBS and subsequently fixed with 4% paraformaldehyde (PFA). A primary monoclonal antibody against TAZ (1:500), FAP (1:50) (Abcam, UK), or Vimentin (1:50) (Santa Cruz) was added and incubated overnight at 4 °C. After washing with PBS for 5 min three times, the secondary antibody Alexa Fluor 488 (Invitrogen Life Technologies, Carlsbad, CA) was applied and further rinsed with PBS for 5 min. Mounting medium VECTASHIELD™ (Vector Laboratory) and DAPI (1:2000) (Roche Biosciences, Indianapolis, IN) were used to counterstain the nucleus. The images were taken with a Carl Zeiss LSM 700 confocal microscope at 630X magnification.

### Electron microscope analysis of exosomes

In brief, a final concentration of 2% PFA was used to fix the exosomes for 2 h. Then, 10 μL of resuspended pellets was adhered to coated EM grids made of Formvar-carbon and incubated in a dry environment for 20 min. The grids were washed with 100 μL PBS before fixation with 1% glutaraldehyde for 5 min. Grids were then rinsed with distilled water and allowed to stand for approximately 2 min for seven more repeats. Uranyl-oxalate solution was applied to the grids for 5 min followed by 50 μL methyl cellulose-UA on ice for 10 min. The overflow fluid was flushed into stainless steel loops to hold the methyl-cellulose film thickness. The grids were dried in air for 5 to 10 min before analyzing by an electron microscope (CM100 Philip) at 80 kV (Department of Electron Microscope Unit, The University of Hong Kong).

### Immunohistochemistry

Fresh tissues were collected and fixed with 4% PFA and stored at 4 °C. Paraffin embedding and tissue sectioning of all the tissues and IHC staining of human tissue were performed by a histopathology service (Department of Pathology, The University of Hong Kong). The primary antibodies for IHC were diluted as follows: αSMA (1:100), FAP (1:100), YAP (1:200), and GROα (1:500). Scoring of the tissue sections was performed by at least two people independently.

### Cytoplasmic and nuclear protein extraction

WPMY-1 scrambled control cells and YAP1 knockdown cells were collected in trypsin-EDTA and centrifuged at 500x g for 5 min. The cells were rinsed with PBS once and centrifuged at 500x g for 2-3 min. The supernatant was removed to collect the cell pellets, and then they were subjected to cytoplasmic and nuclear protein extraction according to the protocols of NE-PER Nuclear and Cytoplasmic Extraction Reagents (78833) (Thermo Science).

### Ex vivo omental-tumor model

The whole omentum was incised from SCID mice and rinsed with PBS. The murine omentum was then placed in a 6-well plate and pretreated with conditioned or normal medium containing 60 ng/mL GROα (Abnova). After 24 h, the omentum was refreshed with a conditioned medium, and GFP-labeled ES-2 GFP/Luc or ID8 GFP/Luc cells were seeded onto the omentum. After 2 weeks of incubation, the omentum was scanned for colonies using fluorescence microscopy (Nikon H550 L).

### Mouse primary stromal cell extraction

Mouse primary stromal fibroblasts were extracted according to reported protocols [30].

### ELISA

A Human CXCL1/GRO-α DuoSet ELISA Kit (DY275-05) was used to validate the cytokines in the conditioned medium of miR-141-overexpressing WPMY-1 cells and scrambled control cells. A total of 100 μL of the sample was applied to each well, and samples were quantified in 6 wells. A mouse CXCL1 ELISA Kit (GRO alpha) (Abcam) was used to validate the cytokines expressed in the conditioned medium of primary stromal fibroblasts from Yap1 cKO mice. For the conditioned medium, the cultured medium was collected after 72 h of incubation with RPMI 1640 medium. The conditioned medium was centrifuged at 1000x g for 5 min to remove cell debris. Wild-type (Yap1^+/+^), heterozygous (Yap1^+/−^), and homozygous (Yap1^−/−^) stromal fibroblast-specific knockouts of Yap1 cKO mice were euthanized to collect whole blood. Approximately 0.5 mL of whole blood per mouse was collected and centrifuged at 2000×g for 15 min to obtain the serum in the supernatant. A total of 50 μL of the sample was analyzed in triplicate according to the manufacturer’s protocol.

### XTT cell viability assay

A Cell Proliferation Kit II (XTT) (Roche Biosciences, Indianapolis, IN) was used to detect cell viability. First, OvCa cells were seeded in 96-well plates at a range of 500-3000 cells per well. After 12-16 hours, the cells were treated with 200 μL of conditioned medium or the treated recombinant protein GROα conditioned with complete medium. Then, 150 μL of XTT mixture (100 μL of PBS, 49 μL of XTT coupling reagent and 1 μL of electron coupling reagent) was added from Day 0 to Day 4 of the treated cells and incubated in 37 °C in the dark for 4 h. The absorbance of each well was measured using a multiwell plate reader (Tecan, Sunrise, Tecan Trading AG, Switzerland). Day 0 was used for normalization, and each treatment was repeated 6 times (*n* = 6).

### Transwell cell migration/invasion assay

Transwell® 6.5 mm inserts with 8.0 μm pore polycarbonate membranes (Corning, NY, USA) and Cell Invasion Kits (Millipore, Billerica, MA) were used for the Transwell cell migration/invasion assay. Ovarian cancer cells were resuspended in FBS-free medium and seeded in the Transwell chamber at a density of 2 × 10^4^ cells per well. A total of 500 μL of conditioned medium from Yap1 cKO mouse stromal fibroblasts or the treated recombinant protein GROα conditioned with the complete medium was added to the lower part of each chamber and incubated for 16-20 h in a 37 °C humidified incubator. The chamber was fixed with 100% methanol for 3 min and stained with 0.5% crystal violet (w/v) for 1 h. Three different fields of each chamber were photographed at random using a microscope (ZEISS) with 400X magnification.

### Syngeneic mouse model of ovarian cancer

A total of 1.0 × 10^7^ ID8 GFP/Luc or ID GFP/Luc Cxcr1 and Cxcr2 CRISPRi cells were injected into the intraperitoneal cavity of 6-20-week-old wild-type (Yap1^+/+^), heterozygous (Yap1^+/−^) and homozygous (Yap1^−/−^) stromal fibroblast-specific knockouts of Yap1 cKO mice. Bioluminescence imaging was performed continuously once a week after the injection. Two hundred μL of D-luciferin (Gold Biotechnology) at a concentration of 15 mg/mL was injected into the intraperitoneal cavity of the mice. After 5 minutes, the mice were anesthetized by intraperitoneal injection of 80-100 (mg/kg) ketamine and 10 (mg/kg) xylazine. The mice were then scanned for bioluminescence using a PE IVIS Spectrum in vivo imaging system (Olympus) in the supine position. Body weights were recorded every week. The mice were euthanized when they met the humane endpoint. The ascites were collected and snap frozen at − 80 °C for ELISA. The tumor tissue was collected and fixed with 4% paraformaldehyde (PFA) and stored at 4 °C for immunohistology.

### Statistical analysis

To quantify the colonies formed in the omentum, the fields of view were selected randomly. ImageJ was used to count the area, numbers of colonies, and quantification of band inetensity of Western blotting. GraphPad Prism was used to perform statistical analysis. Statistical details of the experiments can be found in the figure legends. Data were analyzed using two-tailed unpaired Student’s t-test and are presented as the mean ± SD or mean ± SEM. Cell proliferation curves were analyzed by two-way ANOVA (or mixed model). Kaplan–Meier survival curves were analyzed using the log rank test. Significance was defined as *P < 0.05, **P < 0.01, ***P < 0.001, ****P < 0.0001.

## Results

### miR-141 is an exosomal miRNA derived from ovarian cancer cells

**Fig. 1:**
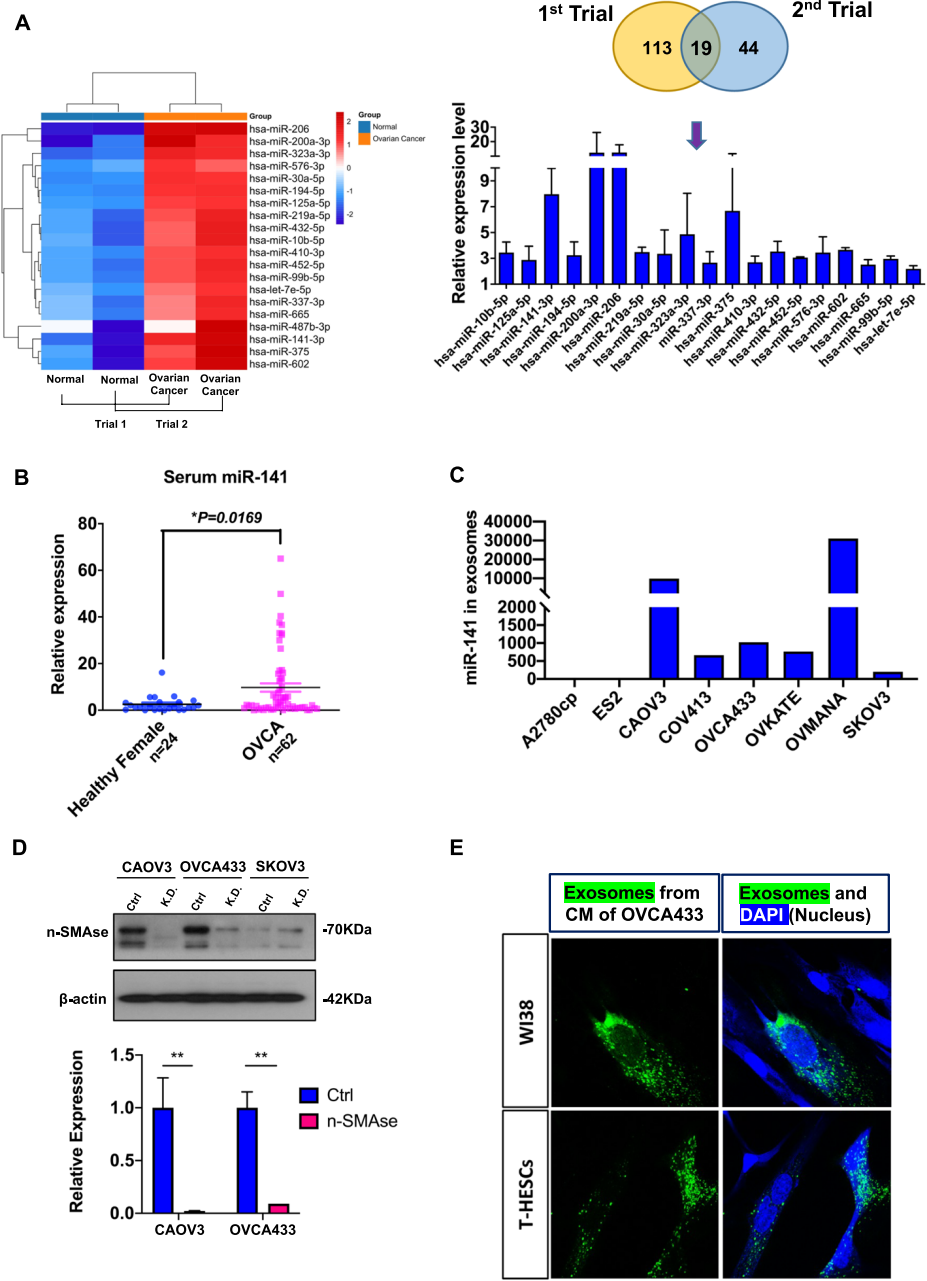
Identification of miR-141 as a secretary miRNA from ovarian cancers. (**A**) The heatmap indicated the changes in secretary miRNA expression in ovarian cancer patients and normal donors using a miRCURY LNA miRNA PCR Array. *N* = 2 independent experiments (left). The graph showed the expression of secretory miRNAs compared between ovarian cancer patients and normal donors in the miRCURY LNA miRNA Cancer Focus PCR Panel (right). N = 2 independent experiments. (**B**) The graphical chart showed the elevated level of exosomal miR-141 in the serum of OvCa patients (*n* = 62) compared with normal women (*n* = 24) by qPCR analysis (mean ± SEM, t-test). (**C**) The graphical chart showed the secretory miR-141 in a panel of ovarian cancer cell lines by qPCR analysis. *N* = 1 independent experiment. (**D**) Knockdown of n-SMase2 by a lentiviral shRNAi approach led to the complete suppression of miR-141 exosome production in the conditioned medium of CAOV3 and OVCA433 cells. β-actin and U6 were the internal controls. (*n* = 3, mean ± SEM, t-test, **P < 0.01) (**E**) The immunofluorescence microscope showed PHK67 labeled exosomal miR-141 derived from OVCA433 conditioned medium, localized in the cytoplasm of WI-38 and T HESC stromal cells. Representative images are shown in color. Blue, DAPI staining in the nucleus. Green, staining of the exosomes with PKH67. Scale bar = 20 μm

To identify the miRNA expression profile in ovarian cancer, the miRNA expression patterns in the peripheral blood samples of 8 OvCa patients (mixed tumor subtypes, tumor grade 1-3, and tumor stage 1-4) compared to eight normal women were analyzed by PCR-based miRNome profiling analysis. After two independent analyses, we found that 19 miRNAs were highly expressed in the peripheral blood samples of OvCa patients (Fig. 1A). Of note, qPCR analysis showed that secreted miR-141 was highly expressed in the serum of OvCa patients compared with healthy females (Fig. 1B), and a panel of human OvCa cells including two HGSOC (OVCA433, and OVKATE), and six human non-HGSOC cell lines (ES-2, SKOV3, A2780cp, CAOV3, COV413, and OVMANA) (Fig. 1C). To elucidate whether the tumor-derived miR-141 mediates cancer-host crosstalk, electron microscopy was firstly showed the existence of exosomes and other small vesicles in the conditioned medium of ovarian cancer cells, indicating the secreted miR-141 might be via an exosomal pathway (Fig. S1A). This postulation was supported by pharmaceutical blockade of exosome production using the nSMase2-specific inhibitor Manumycin-A or genetic depletion of nSMase2, and both treatments remarkably reduced the levels of secreted miR-141 in CAOV3- and OVCA433-conditioned media (Fig. 1D, Fig. S1B). Cy3 labeling of pool exosomes or PKH67-labeled exosomal miR-141 demonstrated that these exosomes, containing miR-141, could mediate cell-to-cell communication in tumor-stromal interactions (Fig. 1E, Fig. S1C).

### MiR-141 induces stromal fibroblasts to have CAF-like properties

**Fig. 2:**
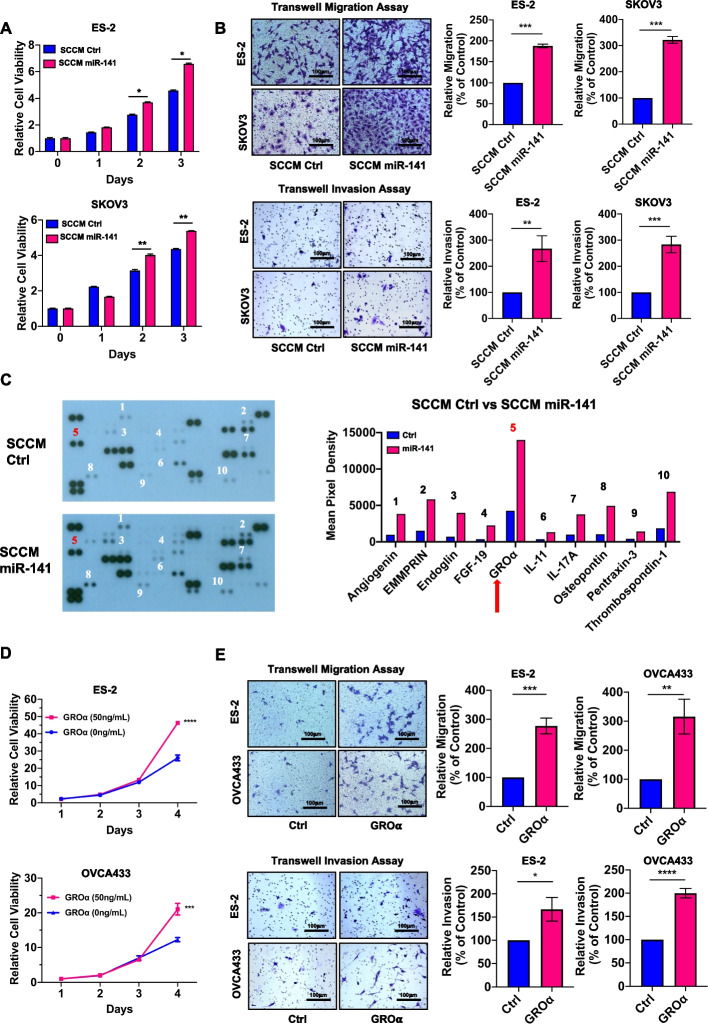
MiR-141 reprograms stromal fibroblasts to be oncogenic drivers, and GROα is a major proinflammatory cytokine (**A**) The XTT cell proliferation assay indicated that stromal cell-conditioned medium (SCCM) from miR-141-transfected WPMY-1 cells (SCCM miR-141) increased the cell proliferation of ES-2 and SKOV3 cells on Day 2 and Day 3, compared with scrambled control medium from WPMY-1 cells (SCCM Ctrl) (*n* = 5, Mean ± SD, t-test, *P < 0.05, **P < 0.01). N = 1 independent experiment. (**B**) Transwell cell migration/invasion assays demonstrated that SCCM miR-141 treatment had convincingly induced a higher capacity of migration at 16 h or invasion at 24 h in ES-2 and SKOV3 cells as compared with SCCM Ctrl treatment (*n* = 6, mean ± SEM, t test, **P < 0.01, ***P < 0.001). Scale bar =100 μm. (**C**) Human XL cytokine array analysis revealed the number of inflammatory cytokines such as GROα in the stromal cell-conditioned medium derived from WPMY-1 scrambled control cells (SCCM Ctrl) and miR-141 overexpressing cells (SCCM miR-141). *N* = 2 independent experiments. (**D**) The XTT cell proliferation assay showed a dose-dependent increase in cell proliferation of ES-2 and OVCA433 cells upon a 4-day incubation with recombinant GROα (50 ng/mL) as compared with the respective untreated control (n = 6, mean ± SEM, two-way ANOVA, **P < 0.01, ***P < 0.001, ****P < 0.0001). *N* = 3 independent experiments. (**E**) Transwell cell migration/invasion assays showed that the recombinant GROα (60 ng/mL) remarkably promoted cell migration/invasion capacity in ES-2 and OVCA433 cells after 14 h (migration) and 20 h (invasion) as compared with the respective untreated control (Ctrl) (n = 6, mean ± SEM, t-test, *P < 0.05, **P < 0.01, ***P < 0.001, ****P < 0.0001). Scale bar = 100 μm

Intriguingly, enforced expression of miR-141 in stromal cells mimicked the effect of exosomal miR-141 in tumor-stromal interactions, and the XTT cell proliferation assay showed that the miR-141 stromal cell conditioned medium (SCCM miR141) could significantly enhance the growth of OvCa cells by approximately 10-40% (Fig. 2A). Furthermore, SCCM miR-141 induced a 2- to 3-fold increase in the rates of migration and invasion of OvCa cells (Fig. 2B). To determine whether miR-141 induces other cell types, a panel of cell lines, including T HESCs, WI-38 and WPMY-1 cell lines, an adipocyte (3 T3-L1) cell line, an endothelial cell line (HUVEC), skin fibroblasts (BJ), Jurkat T cells, and a human epithelial kidney cell line (HEK293), was used to induce the expression of miR-141 (Fig. S2A). The XTT cell proliferation assay showed that the CM of stromal cells (T HESCs, WI-38 cells, and WPMY-1 cells), but not HEK293 cells, BJ skin fibroblasts, Jurkat T cells, 3 T3-L1 adipocytes, or HUEVC endothelial cells, could promote OvCa cell growth (Fig. S2B), supporting our notion that miR-141 converting stromal cells to tumor-promoting CAFs is highly cell context- or cell type-dependent.

### GROα is the major proinflammatory chemokine in miR-141-expressing stroma

Proinflammatory cytokines are critical oncogenic promoting factors in tumor progression and metastasis in both autocrine and paracrine manners [31, 32]. To determine which cytokines are the main oncogenic drivers in the SCCM miR-141, a Proteome Profiler Human XL Cytokine Array was performed, and the data indicated that GROα was the pivotal oncogenic driver in the SCCM miR-141 as compared with SCCM Ctrl (Fig. 2C). Consistent with the above findings of miR-141 in stromal cells, the level of *GROα* was merely upregulated in stromal cells but not in OvCa cells, which endogenously and highly upregulated miR-141 according to our previous report [28] (Fig. S2C). Since patients with advanced OvCa usually have omental metastasis [33], an OvCa cancer cell-murine omental co-culture system was consequently designed to mimic ex vivo tumor colonization. Given the similarities between human and murine miR-141 in function [34, 35], our study showed that the level of *GROα* in omental tissues increased 2.5-fold (Fig. S2D). XTT cell proliferation assays showed that OVCA433 and ES-2 cells cotreated with GROα recombinant protein had significantly increased the cell growth rate as compared with their untreated controls (Fig. 2D). Likewise, human recombinant GROα enhanced the migration and invasion of ovarian cancer cells to various degrees (Fig. 2E). Indeed, ELISA results showed that GROα was at least 2-fold higher in the cancerous omental conditioned medium (OCM) (*n* = 10) than in the normal OCM (*n* = 6) (Fig. S2E). This finding showed that the proinflammatory GROα possesses oncogenic promoting effects and is highly expressed in the TME of ovarian cancers.

### YAP1 of hippo signaling is the direct target of miR-141

LC-MS/MS proteomic profiling was utilized to analyze how miR-141 modulated stromal cells to produce proinflammatory cytokines. Analytical findings indicated that YAP1 is the putative target of miR-141 in WPMY-1 stromal cells (Fig. S3A). Western blot analysis showed that YAP1 was downregulated in primary CAFs isolated from OvCa patients as compared with primary endometrial stromal fibroblasts (Fig. S3B).

**Fig. 3:**
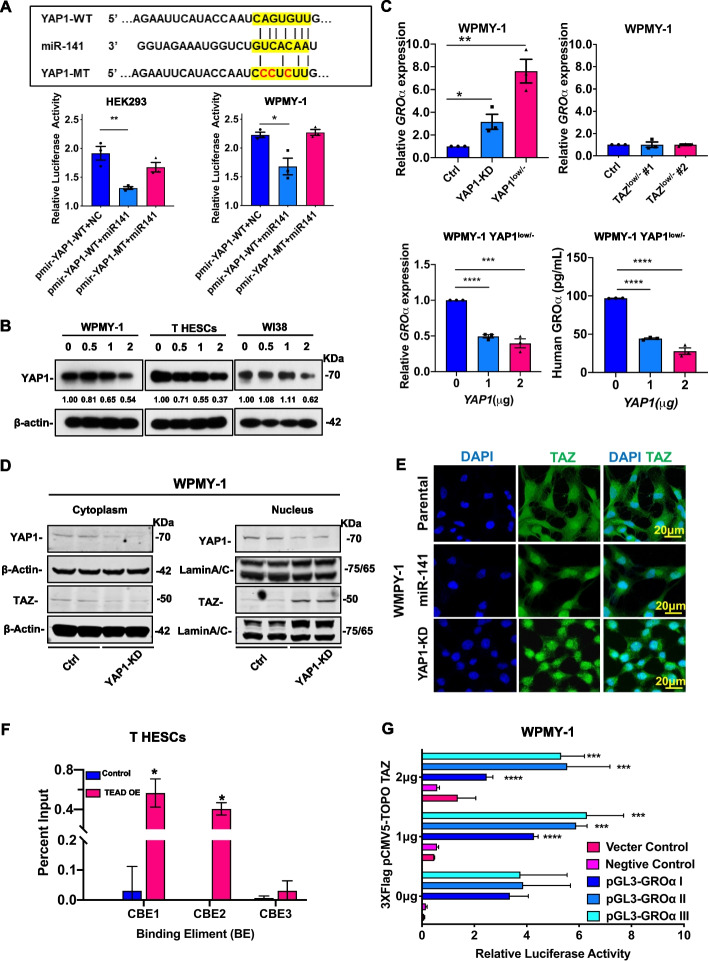
YAP1 of Hippo signaling is the direct target of miR-141 (**A**) The schematic drawing showed YAP1 constructs with wild-type (YAP1-WT) and mutant (YAP1-MT) miR-141 binding sites paired with the miR141 sequence (upper). Dual-luciferase assay showed the relative luciferase activities that cotransfection of pmir-YAP1-WT or pmir-YAP1-MT and the miR-141 expressing plasmid pmR-141 (pmR-ZsGreen1 empty vector was used as negative control) in WPMY-1 and HEK293 cell lines (mean ± SEM, t-test, *P < 0.05, **P < 0.01). *N* = 3 independent experiments. (**B**) Confirmation of miR-141 as a target of YAP1 by dose-dependent transfection of pmR-Zsgreen1-miR141 (0, 0.5, 1.0, and 2.0 μg) into WPMY-1, T HESC and WI-38 cells by western blot analysis. The relative YAP1 expression (YAP1/β-actin) was quantified by ImageJ software. (**C**) Graphic charts compared the relative transcription level of *GROα* between WPMY-1 stromal cells with either shRNA-mediated knockdown of YAP1 (YAP1-KD), CRISPR/Cas9 system-mediated knockout of YAP1 (YAP1^low/−^) or knockout of TAZ (TAZ^low/−^ #1 and TAZ^low/−^ #2) with the respective control (Ctrl) (upper). QPCR and ELISA analyses showed the relative expression of GROα in WPMY-1 YAP1^low/−^ cells transfected with the YAP1-expressing plasmid (0, 1 and 2 μg) (lower) (mean ± SEM, t-test, *P < 0.05, **P < 0.01, ***P < 0.001, ****P < 0.0001). N = 3 independent experiments. (**D**) Western blot analysis showed the changes in YAP1/TAZ in the cytoplasm and nucleus in WPMY-1 cells with shRNA-mediated YAP1 knockdown approach (YAP1-KD). β-actin and Lamin A/C were used as the internal controls for the cytosol and nuclear proteins, respectively. (**E**) Immunofluorescence microscopy showed changes in YAP1/TAZ in the cytoplasm and nucleus in YAP1 knockdown and miR-141-overexpressing WPMY-1 cells, and parental cells were used as a control. Scale bar = 20 μm. (**F**) The graph showed the relative percent input of ChIP that indicated the interaction between TEAD and the predicted binding sites on GROα in T HESCs cells with TEAD overexpression and vector control (mean ± SEM, t-test, *P < 0.05). (**G**) Graphic charts show the relative luciferase activity of cotransfected TEAD1, TAZ and pGL3-GROα (N ~ III) in the WPMY-1 cells by dual-luciferase assay (mean ± SEM, one-way ANOVA, *P < 0.05, ***p < 0.001, ****p < 0.0001). *N* = 3 independent experiments

Given the existence of complementary binding sites between miR-141 and the 3′-UTR of YAP1, a dual-luciferase reporter assay was performed to verify whether YAP1 is the direct target of miR-141 (Fig. 3A). The results showed that miR-141 could significantly cause 50-60% reduction in the luciferase signals in the wild-type (pmir-YAP1-WT) but not in the mutant (pmir-YAP1-MT) of YAP1 3’UTR (Fig. 3A). Western blot analysis showed that transient transfection of miR-141 caused a remarkable reduction of YAP1 in stromal fibroblasts (Fig. 3B).

Numerous studies have proposed that YAP1 and its paralog TAZ function as either a coactivator or corepressor in different cell contexts [36, 37]. The results herein revealed that shRNA-mediated knockdown or CRISPR/Cas9-mediated knockout of YAP1 in WPMY-1 stromal cells led to an increased transcriptional expression of *GROα*, whereas overexpression of YAP1 markedly reduced the levels of *GROα* (Fig. 3C, Figs. S3C and D). In addition, no apparent induction of *GROα* occurred in clones of WPMY-1 cells (TAZ^low/−^ #1 and TAZ^low/−^ #2) with CRISPR/Cas9-mediated gene knockout of TAZ respectively (Fig. 3C, Fig. S3C).

In line with these findings, IHC analysis revelated that the downregulated YAP1 (~ 2-fold) was inversely correlated with the increased GROα (~ 5-fold) level in the cancerous stroma compared with the normal stroma in a commercial OvCa tissue array (*n* = 12 pairs of cancerous and normal tissues, OV241C, Biomax) and OvCa clinical samples (*n* = 14 OvCa and *n* = 10 normal endometrial tissues) (Figs. S3E and F). These outcomes collectively confirmed that downregulated YAP1 is associated with GROα production and can be observed clinically.

The nuclear localization of YAP1 and its paralog TAZ is a critical event in regulating gene expression through interaction with the TEA domain family transcription factors TEAD1-4 [38, 39]. Western blot analysis showed that knockdown of YAP1 in WPMY-1 stromal cells led to enhanced localization of TAZ within the nucleus (Fig. 3D). Immunofluorescence microscopy similarly revealed that the depletion of YAP1 or enforced expression of miR-141 induced more nuclear TAZ accumulation in WPMY-1 stromal cells (Fig. 3E). To examine whether TAZ and TEAD1 could transcriptionally activate GROα in stromal cells, the TEAD1 motif was used to scan the putative TEAD1-binding sites on *GROα* (*CXCL1*) promoters (−2kbp ~ +2kbp) according to our previous report [40], and three direct binding sites of TEAD1 on the *GROα* promoters (CBE1-3) were found (Figs. S4A and B, Table S1). Chromatin immunoprecipitation (ChIP) and Dual-luciferase assay (DLA) analyses demonstrated that TEAD1 had the most potent binding capacity on CBE1 of the *GROα* promoter (Fig. 3F and G). These outcomes support the finding that YAP1 is a corepressor of the transcriptional regulation of the TAZ/YAP1/TEAD transcriptional complex in stromal cells (Figs. S4A and B).

### GROα enhances tumor colonization of ovarian cancer cells

Given that GROα released from stromal cells could significantly promote the proliferation and aggression of ovarian cancer cells, an ex vivo murine omentum-cancer cell co-culture system was employed to demonstrate whether recombinant GROα proteins could exert similar growth promoting effects on OvCa cells. Fluorescence microscopy revealed that the number and size of the colonies formed by both the GFP-labeled human OvCa cell line ES-2 cells (ES-2 GFP) and the murine OvCa cell line ID8 cells (ID8 GFP) on the murine omentum were markedly increased upon treatment with recombinant GROα (Figs. S4C and D).

**Fig. 4:**
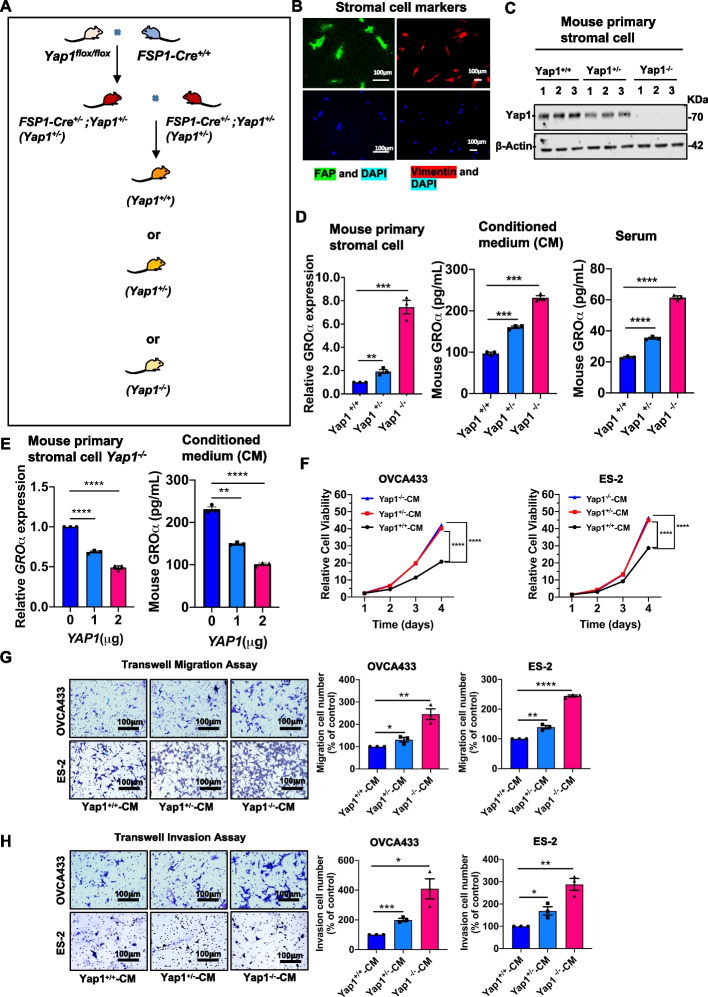
GROα enhances the tumor colonization of ovarian cancer cells (**A**) The graph showed the procedures for generating Yap1 stromal-specific cKO mice by crossing Yap1^flox/flox^ mice with FSP1-Cre^+/+^ mice. Three genotypes of mice (Yap1^+/+^,  Yap1^+/−^, and Yap1^−/−^), were generated. (**B**) Immunofluorescence microscopy showed the mouse stromal fibroblasts extracted from Yap1 stromal-specific cKO mice (Yap1^+/−^ and Yap1^−/−^) stained by the stromal cell markers FAP (green) and vimentin (red). Scale bar = 100 μm. (**C**) Western blot analysis compared the expression of Yap1 in mouse stromal fibroblasts extracted from Yap1 stromal-specific cKO mice (Yap1^+/−^ and Yap1^−/−^) with control of wild-type (WT) mice (Yap1^+/+^). (**D**) Bar charts showed the relative expression level of GROα in mouse stromal fibroblasts, murine stromal conditioned medium, and serum extracted from Yap1^+/+^, Yap1^+/−,^ and Yap1^−/−^ mice by qPCR analysis and ELISA (*n* = 3, mean ± SEM, t-test, **P < 0.01, ***P < 0.001, ****P < 0.0001). (**E**) qPCR analysis and ELISA showed the relative expression of GROα in mouse stromal fibroblasts and murine stromal conditioned medium from Yap1^−/−^ mice that were transiently transfected with YAP1-expressing plasmid (0 μg, 1 μg and 2 μg) (n = 3, mean ± SEM, t-test, **P < 0.01, ****P < 0.0001). (**F**) The XTT cell proliferation assay indicated the relative cell growth of OVCA433 and ES-2 cells co-cultured with mouse stromal conditioned medium derived from Yap1 stromal-specific cKO mice (Yap1^+/−^-CM and Yap1^−/−^-CM). Stromal conditioned medium derived from Yap1^+/+^ mice was used as a control (*n* = 6, mean ± SEM, 2-way ANOVA, ****P < 0.0001). *N* = 3 independent experiments. (**G**) The Transwell migration assay showed that the murine stromal conditioned medium from Yap1 cKO mice (Yap1^+/−^-CM and Yap1^−/−^-CM) promoted OVCA433 and ES-2 migration capacities, as compared with the respective control treated with Yap1^+/+^-CM (*n* = 3, mean ± SEM, t-test, *P < 0.05, **P < 0.01, ****P < 0.0001). N = 3 independent experiments. (**H**) The Transwell invasion assay showed that the murine stromal conditioned medium from Yap1 cKO mice (Yap1^+/−^-CM and Yap1^−/−^-CM) promoted OVCA433 and ES-2 invasion capacities, as compared with the respective control treated with Yap1^+/+^-CM (n = 3, mean ± SEM, *P < 0.05, **P < 0.01, ***P < 0.001). *N* = 3 independent experiments

Next, to validate whether the downregulated YAP1 rewires stromal cells for enhanced production of stimulatory growth factors such as GROα to form a premetastatic niche in vivo, stromal-specific Yap1 conditional knockout (cKO) mice bearing two loxP sites flanking the exons of the Yap1 gene were crossbred with FSP-Cre mice to selectively eradicate Yap1 expression in stromal fibroblasts, resulting in Yap1^+/−^ and Yap1^−/−^ mice (Fig. 4A). The knockout efficiency of Yap1 in the marker-verified primary stromal cells from Yap1 cKO mice was confirmed by genotyping (data not shown) and Western blot analysis (Figs. 4B and C). As expected, qPCR analysis showed that *GROα was* drastically upregulated in primary stromal cells isolated from Yap1^+/−^ and Yap1^−/−^ mice compared with wild-type (WT) Yap1^+/+^ mice (Fig. 4D). ELISA results confirmed that GROα was highly upregulated by ~ 80% and ~ 130% in CM generated from primary stromal cells of Yap1^+/−^ and Yap1^−/−^ mice, respectively (Fig. 4D). Additionally, a similar incremental pattern of GROα was observed in the serum of the Yap1^+/−^ and Yap1^−/−^ mice (Fig. 4D). Additionally, rescue experiments with overexpression of Yap1 markedly reduced the levels of *GROα* in primary stromal cells from Yap1^−/−^ mice and the corresponding CM generated (Fig. 4E). These findings substantiated the claim that the depletion of Yap1 leads to the enhanced production of GROα in stromal cells.

Since YAP1-defective stromal cells have increased potential for GROα production, it is worth further verifying whether CM from Yap1-deficient stromal cells promotes the oncogenic properties of OvCa. The XTT cell proliferation assay showed a 2- to 3-fold increase in cell growth in OvCa cells upon co-culture with CM from the primary stromal cells of Yap1^+/−^ and Yap1^−/−^ mice (Fig. 4F). Transwell cell migration and invasion assays likewise demonstrated that the numbers of migrated and invaded OvCa cells increased 1.3- to 2-fold and 1.69- to 4-fold, respectively, upon treatment with CM from the primary stromal cells of Yap1^+/−^ and Yap1^−/−^ mice (Figs. 4G and H). Taken together, these findings suggest that GROα in the CM of Yap1-depleted stroma is responsible for facilitating tumor colonization of OvCa cells, and GROα exerts a relatively stronger oncogenic potential to OvCa cells.

### Conditional depletion of Yap1 in the stroma promotes in vivo tumor dissemination

**Fig. 5:**
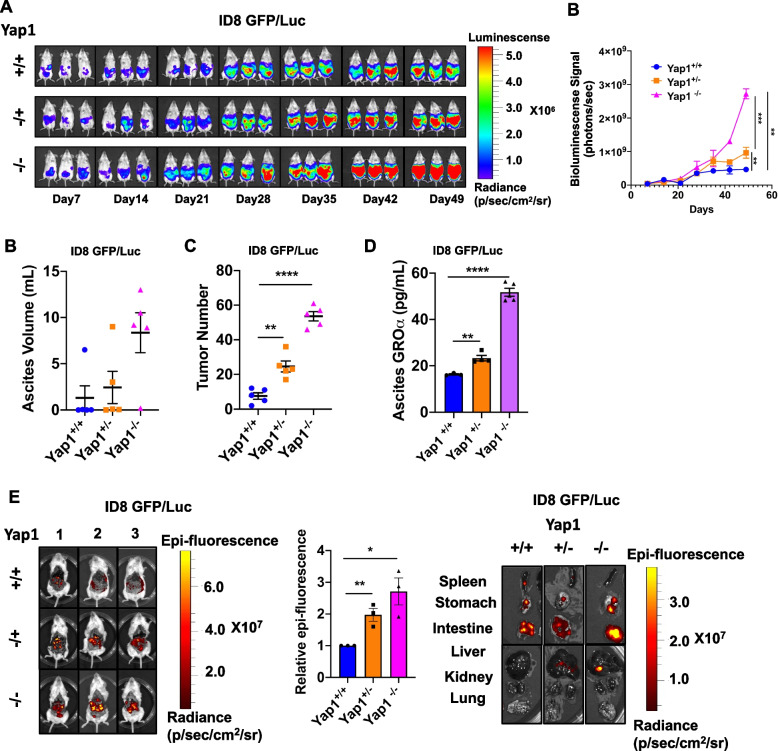
Conditional knockout (cKO) of Yap1 in the stroma promotes in vivo tumor dissemination (**A**) The images (left) and quantifications (right) of the bioluminescence signals among Yap1^+/+^, Yap1^+/−^ and Yap1^−/−^mice upon intraperitoneal injection of GFP/Luc-labelled ID8 cells from Day 7 to Day 49 (*n* = 3, mean ± SEM, 2-way ANOVA, **P < 0.01, ***P < 0.001). (**B**) Dot plots displayed the ascites volume among Yap1^+/+^, Yap1^+/−,^ and Yap1^−/−^ mice with an intraperitoneal injection of GFP/Luc-labelled ID8 cells (*n* = 5, mean ± SEM). (**C**) Dot plots displayed the number of tumor nodules among Yap1^+/+^, Yap1^+/−,^ and Yap1^−/−^ mice with an intraperitoneal injection of GFP/Luc-labelled ID8 cells (n = 5, mean ± SEM, t-test, **P < 0.01, ****P < 0.0001). (**D**) ELISA revealed the amount of GROα in the ascites of Yap1^+/+^, Yap1^+/−^ and Yap1^−/−^ mice with an intraperitoneal injection of GFP/Luc-labelled ID8 cells (*n* = 3, mean ± SEM, t-test, **P < 0.01, ****P < 0.0001). (**E**) The images (left and right) and quantifications (middle) of the intraperitoneal epifluorescence signals among Yap1^+/+^, Yap1^+/−^ and Yap1^−/−^ mice with an intraperitoneal injection of GFP/Luc-labelled ID8 cells (n = 3, mean ± SEM, t-test, * *P* < 0.05, **P < 0.01)

As the augmented GROα in the CM of Yap1-depleted stromal cells significantly exerted in vitro efficacy in promoting the progression and aggression of ovarian cancer cells, it is of interest to determine whether GROα also stimulates in vivo tumor dissemination in a syngeneic mouse model of ovarian cancer. To this end, ID8 cells with GFP/Luc were intraperitoneally injected into WT mice as well as Yap1^+/−^ and Yap1^−/−^ mice, and tumor growth was evaluated by bioluminescence imaging (BI) (Fig. 5A). The results showed that tumor formation was more prevalent in Yap1^+/−^ and Yap1^−/−^ mice than in WT mice (Fig. 5A, Fig. S5A-C). In particular, the tumor growth rate, the number of tumor nodules, and the volume of ascites in Yap1^−/−^ mice were approximately 4.5-fold greater than those in either WT or Yap1^+/−^ mice (Figs. 5A-C). Of note, the concentration of GROα in the ascites of Yap1^−/−^ mice was 2- to 3-fold greater than that of WT or Yap1^+/−^ mice (Fig. 5D), suggesting that GROα is relatively dominant as a potential tumor-promoting chemokine. Consistent with the outcomes of BI, fluorescent imaging revealed the strongest signal of epifluorescence in the intraperitoneal cavity and the viscera (spleen, stomach, intestine, liver, lung, etc.) of the Yap1^−/−^ mice compared with the WT and Yap1^+/−^ mice, indicating that the intraperitoneal microenvironment of Yap1^−/−^ mice facilitated a higher tumor formation ability and metastatic potential of OvCa cells (Fig. 5E).

### Depletion of CXCR1/2 impairs the oncogenic potential of OvCa cells

**Fig. 6:**
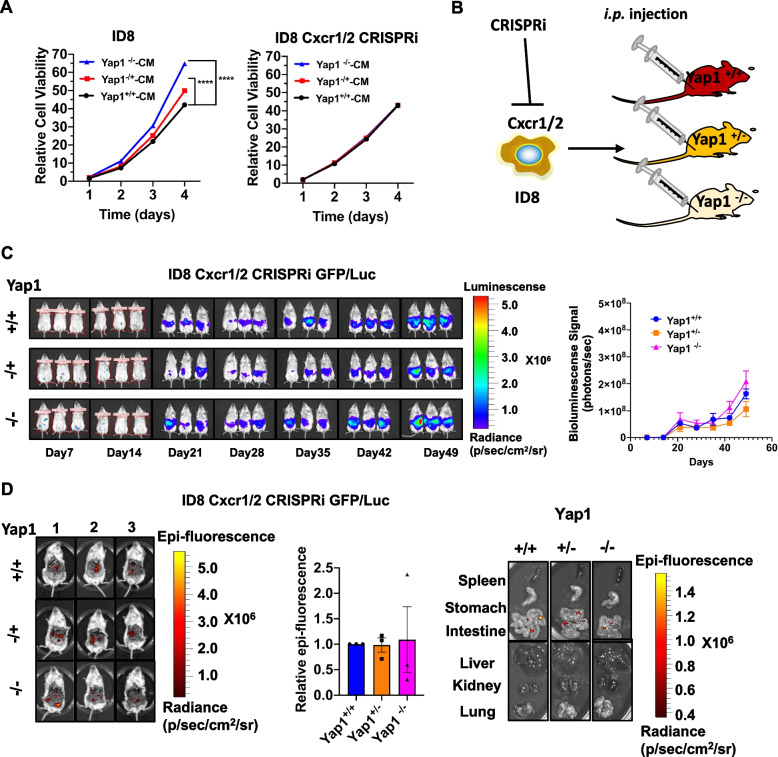
Depletion of CXCR1/2 impairs the oncogenic potential of OvCa cells (**A**) XTT cell proliferation assay indicated the relative cell growth of ID8 and ID8 Cxcr1/2 CRISPRi cell lines when co-cultured with murine stromal conditioned medium from Yap1 stromal-specific cKO mice (Yap1^+/−^-CM and Yap1^−/−^-CM). Yap1^+/+^-CM was used as a control (*n* = 6, mean ± SEM, 2-way ANOVA, ****P < 0.0001). N = 3 independent experiments. (**B**) The schematic diagram showed the workflow of how ID8 cells with Cxcr1/2 were silenced using CRISPR interference (CRISPRi) approach (ID8 Cxcr1/2 CRISPRi cells) were generated and intraperitoneally injected into Yap1^+/+^, Yap1^+/−^ and Yap1^−/−^ mice to establish a mouse tumor model. (**C**) The images (left) and quantifications (right) of the bioluminescence signals among Yap1^+/+^, Yap1^+/−^ and Yap1^−/−^ mice with an intraperitoneal injection of GFP/Luc-labelled ID8 Cxcr1/2 CRISPRi cells from Day 7 to Day 49 (n = 3, mean ± SEM). (**D**) The images (left and right) and quantifications (middle) of the intraperitoneal epifluorescence signals among Yap1^+/+^, Yap1^+/−^ and Yap1^−/−^ mice with an intraperitoneal injection of GFP/Luc-labelled ID8 Cxcr1/2 CRISPRi cells (n = 3, mean ± SEM)

Mounting evidence suggests that G-protein-coupled receptors (GPCRs), such as CXC chemokine receptor 1/2 (CXCR1/2), are the mutual receptors of GROα in human cancers, including ovarian tumors [41–43]. Aberrant upregulation of CXCR1/2 is attributed to augmented tumor progression in ovarian cancers, evincing that GROα mediates the oncogenic capacities of cancer invasion and migration by interacting with CXCR1/2 in OvCa [42, 44, 45]. To examine whether inhibiting CXCR1 or CXCR2 in OvCa cells could reverse the oncogenic promoting effect of GROα, CRISPR interference (CRISPRi) was used to silence murine Cxcr1/2 in ID8 cells (Fig. S6A). The deletion of Cxcr1/2 did not alter the growth rate of ID8 cells (Fig. S6B). As expected, recombinant GROα failed to promote the cell growth rate in ID8 Cxcr1/2 CRISPRi cells (Fig. S6C) Likewise, CM from both the primary stromal cells of Yap1^+/−^ and Yap1^−/−^ mice lost the ability to enhance cell proliferation of Cxcr1/2-depleted ID8 cells (Fig. 6A). More importantly, the cell migration/invasion capacities of ID8 Cxcr1/2 CRISPRi cells were completely impaired even upon treatment with CM from the stromal cells of Yap1^+/−^ and Yap1^−/−^ mice (Figs. S6D and E). Consistently, the tumor growth and metastatic potentials of ID8 Cxcr1/2 CRISPRi cells with GFP/Luc were remarkably reduced in the WT mice as well as the Yap1^+/−^ and Yap1^−/−^ mice without any apparent change in survival and body weight (Figs. 6B-D, and Figs. S5D-G). These data collectively indicate that GROα is a key cytokine involved in promoting tumor dissemination of OvCa cells. In contrast, depletion of its chemokine receptors CXCR1/2 on OvCa cells restrains the oncogenic potential mediated by the GROα-enriched TME during OvCa peritoneal metastasis.

## Discussion

Investigating the mechanisms of disseminated tumor cells with a preference for particular distal sites for metastatic seeding has become a hot topic in cancer metastasis research [10, 46]. Recent investigations have depicted that exosomes with secreted miRNAs are vital constituents of the secretome that participated in the oncogenic reprogramming of cancer cells and forming premetastatic niches [47, 48]. Here, we reported that miR-141 is a highly expressed exosomal miRNA from the secretome of OvCa cells that tends to reprogram stromal fibroblasts to form a permissive niche by altering Hippo/YAP1 signaling to secrete metastasis-promoting cytokines. Using in vitro, ex vivo*,* and in vivo stromal-specific Yap1 cKO mouse models combined with clinical relevance data, we demonstrated that stromal cells could be markedly remodeled by tumor-derived miR-141 to show CAF-like capacities that scaled up the production of the proinflammatory chemokine GROα, which in turn, promoted metastatic colonization in ovarian cancers. Noticeably, depletion of Cxcr1/2 receptors impeded the oncogenic effects of GROα, preventing intraperitoneal dissemination of ovarian tumors and highlighted the possibility of targeting the miR-141/YAP1/GROα/CXCR1/2 signaling cascade to combat OvCa metastases.

Consistent with the ‘seed and soil’ hypothesis put forward by Stephen Paget, metastatic tumor cells often exhibited organ tropism during malignant metastasis that they displayed preferential tumor colonization on specific secondary organs depending on the types of neoplasm. The stromal cells that form the epithelial basement membrane and produce the extracellular matrix (ECM) [49] are required in the dynamic interactions of the TME with malignant cells [50]. To this end, it is important to focus on the underlying mechanism by which stromal cells are reprogrammed and transmuted to CAFs by tumor-derived exosomal miRNAs. PCR-based miRNome profiling analysis was thus performed on peripheral blood samples from a group of OvCa patients with mixed tumor subtypes, stages, and grading. Recent evidence has suggested that miR-141 promotes metastatic progression, and the upregulated circulating miR-141 in the blood serum is significantly associated with advanced EOC tumor progression [28, 51, 52]. Hence, by the rigorous testing miR-141 by the stromal cell-based functional assays, miR-141 showed a relatively higher potential in remodeling stromal cells into CAFs phenotype, and importantly, its expression in blood serum was strongly supported by the clinical evidence. On the other hand, circulating miRNAs derived from tumors are shielded from decomposition using inclusion in RNA binding proteins or exosomes [53]. However, only exosomes played a decisive role in intercellular communication and transferring the enclosed miRNAs for altering recipient stromal cells to form a pre-metastatic niche during metastatic colonization [54]. To this end, we performed pharmacological and genetic inhibition of the exosomal key factor nSMase2 [55], and immunofluorescence microscopy analysis using Cy3-miR-141 or PKH67-labeled exosomes confirmed that the circulating miR-141 is derived from OvCa cells via the exosomal pathway, suggesting miR-141 possesses the capacity of mediating cell-to-cell communication in tumor-stroma interactions.

MiR-141 is one of the members of the miR-200 family, which has been known to critically regulate and play a biphasic role in modulating epithelial-mesenchymal transition (EMT) and mesenchymal-epithelial transition (MET) of epithelial cancer cells during metastases [56]. Unlike other human cancers, miR-200 family members are reproducibly upregulated in advanced ovarian cancers during cancer progression due to frequent genomic amplification and/or chromosomal gains [57, 58]. We have recently reported that upregulated miR-141 enhances anoikis resistance in metastatic progression of OvCa [28]. In addition, miR-141 has been associated with cisplatin resistance [59] and increased tumor growth of OvCa under oxidative stress [60]. Each piece of evidence indicates that the multifunctional roles of the miR-200 family are determined by the content and cell plasticity of cancer cell types. In this study, we further reported that exosomal miR-141 lowers nuclear YAP1 but enhances TAZ/TEAD1 transcriptional activity to produce GROα, as observed in CAFs of OvCa patients. Interestingly, exosomal miR-141 could only reprogram stromal cells but not other cell types, including miR-141-overexpressing OvCa cells, suggesting the effect of miR-141-mediated cell reprogramming is highly cell context- or cell type-dependent.

YAP1 and TAZ are the two vital downstream effectors of the Hippo signaling cascade and closely interact with one another through the PDZ domains [61]. TAZ has been regarded as a paralog of YAP1. Nevertheless, recent evidence suggests that the complexity of YAP1/TAZ is more significant than expected [62]. Our findings revealed that the reduction of YAP1 by either miR-141 or specific knockdown resulted in the augmentation of *GROα* in stromal cells, while restoration of YAP1 expression significantly attenuated the upregulation of *GROα.* Numerous studies have shown that YAP1 and TAZ function as transcription coactivators and corepressors by interacting with the TEAD transcription factor family to modulate the expression of different genes, including proinflammatory cytokines and chemokines, depending on the cell context [36, 37, 63, 64]. In cancers, TAZ, but not YAP1, acts as an oncogene to promote cancer progression [64]. Both upstream and downstream regulation of miR-141 and Hippo pathway have been demonstrated in many cancers. In esophageal squamous cell carcinoma, ectopic expression of miR-141 directly inhibits the YAP1-mediate cisplatin-sensitive pattern of cancer cells [65]. It was also reported that miR-141 could be counteracted by long noncoding RNA (lncRNA) HCG18, therefore, the YAP/TAZ expression level was upregulated and promoted tumor cell migration and invasion in gastric cancer [66]. The prognostic value of the miR-141/YAP1 pathway was also proved by analyzing the association between miR-141 expression and clinical outcomes in pancreatic ductal adenocarcinoma patients [67]. However, the functional roles and molecular mechanisms of Hippo/YAP1 signaling in stromal cells or CAFs remain obscure. The study herein revealed that the GROα promoter has three TEAD1 binding sites. Therefore, miR-141 directly targets YAP1 in stromal cells and prompts TAZ to translocate from the cytoplasm to the nucleus, which triggers the TAZ/TEAD1 transcriptional complex binding and promotes the transcription of *GROα* in stromal cells. This suggests that YAP1 in stromal cells acts as a repressor in modulating TAZ/TEAD1-dependent transcriptional activities and that GROα is one of the vital transcriptional products based on our findings. However, further verification is warranted.

Chronic inflammation is highly correlated to human cancer development [68–70]. Akin to other solid tumors, the progression of OvCa is unambiguously associated with inflammation and a complicated network of chemokines [70–72]. Chemokines are a family of small cytokines or signaling factors released from cells that induce chemotaxis in nearby responsive cells. Increasing evidence has demonstrated that the enriched secretion of chemokines, such as GROα, from CAFs, contributes to progression and poor prognosis in breast cancer [73]. Furthermore, peritoneal inflammation is usually observed in advanced OvCa [71]. Transported by the peritoneal fluid, cell spheroids of OvCa could attach preferentially to the omentum with constitutive production of inflammatory cytokines [74]. However, the significance of GROα in boosting the metastatic progression of OvCa is poorly understood. In this study, in vitro and ex vivo functional experiments demonstrated the oncogenic effects of GROα in promoting cell proliferation, cell migration/invasion, and colony formation. As previously mentioned, it is postulated that miR-141 acts as an oncogenic miRNA by targeting YAP1 in stromal cells to form a premetastatic niche. To verify this hypothesis, a stromal Yap1 cKO mouse model was utilized to mimic the TME of OvCa peritoneal metastasis. As expected, murine GROα in the circulation, including the stromal fibroblasts and the sera of both heterogeneous and homogenous stromal-specific Yap1 cKO mice, was significantly elevated, as we observed clinically. Functional assays also confirmed the oncogenic potential of GROα in the conditioned medium of Yap1 cKO stroma, suggesting that tumor-derived miR-141 remodels stromal cells to become CAFs, which secrete GROα to promote tumor colonization during OvCa metastasis.

Given that the elevation of proinflammatory chemokines is attributed to the loss of YAP1 signaling mediated by miR-141, the direct and practical approach to reducing their levels in the TME is using anti-microRNA (anti-miRs) to target exosomal miR-141 or to restore YAP1 repression on the TAZ/TEAD1 transcriptional complex. However, the lack of commercial reagents makes these methods infeasible. Alternatively, blocking the CXCR1/2 receptors of the dominant proinflammatory chemokine GROα is another probable therapeutic approach [75–77]. Actually, CXCR1/2 inhibitors per se or in combination with other therapeutics have exhibited the significant potential to restrain the progression of OvCa. For example, Le Naour et al. found that AS-62401, a CXCR1/2 inhibitor, could block the tumor-promoting function of these receptors and sensitize the OvCa cell to platinum treatment [78]. Furthermore, the combination of CXCR2 inhibitor SB225002 with Sorafenib significantly yielded synergetic antitumor and anti-angiogenesis responses in OvCa [79]. Several small-molecule CXCR1/2 inhibitors also presented potent therapeutic efficacy upon combined use with other targeted therapies, chemotherapies, and immunotherapies in various malignancies such as head and neck squamous cell carcinoma, non-small cell lung cancer, prostate cancer, and breast cancer [41, 80–83]. A window-of-opportunity study on breast cancer also showed that Reparixin, an oral tablet of CXCR1/2 inhibitor, reduced the cancer stem cells (CSCs) content and appeared safe and well-tolerated among the treated patients [84]. In this study, our data consistently showed that GROα is a crucial chemokine in OvCa metastatic colonization based on its expression levels. Indeed, GROα is a protein structurally related to interleukin-8 (IL-8), and CXCR1/2 receptors are the specific receptors of GROα/IL8 [85, 86]. Recent evidence has suggested that CXCR1/2 inhibitors can be utilized to prevent OvCa cells from the protumoural effects of CAFs [78]. In addition, harnessing a monoclonal antibody, Y3041658, for effective antagonization of human CXCR1/2 is also a promising strategy to impair GROα-mediated tumor-promoting effects [87]. In this study, CRISPRi technology was utilized to inhibit the expression of Cxcr1/2 receptors simultaneously in the murine high-grade serous OvCa cell line, ID8 cells. The deletion of Cxcr1/2 did not alter the growth rate of ID8 cells but caused the loss of the tumor-promoting effect on cancer cell dissemination and progression in the GROα-enriched TME. These findings have suggested not only the significance of the miR-141/Hippo/YAP1/proinflammatory chemokine signaling cascade in the establishment of a premetastatic niche, but also raised the possibility of targeting this signaling axis by using inhibitors or monoclonal antibodies against CXCR1/2 in OvCa cells as a novel therapeutic intervention for preventing the metastatic progression of OvCa.

## Conclusions

We illustrated that the contribution of elevated miR-141, an OvCa-derived exosomal microRNA, is related to the progression of OvCa and that the inner mechanism of miR-141 remodels stromal cells to tumor-promoting phenotypes. Based on this study, we propose that YAP1, a repressor of the YAP1/TAZ/TEAD1 transcription complex, is directly regulated by exosomal miR-141 in stromal cells and subsequently elevates the production of GROα, which is abundant in the premetastatic niche of OvCa. GROα is one of the oncogenic chemokines promoting cell proliferation, cell migration/invasion, and ex vivo tumor colonization of OvCa cells. The simultaneous silencing of the GROα receptors CXCR1/CXCR2 on OvCa cells with CRISPRi technology reduces the oncogenic promoting effects mediated by GROα, providing new insight into therapy for OvCa patients. Future prospective new therapies for OvCa could concentrate on monoclonal antibodies targeting GROα receptors or CRISPR gene editing techniques based on eukaryotic cells.

## Supplementary Information

**Additional file 1: Supplementary Table S1.** Oligo sequences of plasmid cloning and qPCR.

**Additional file 2: Supplementary Table S2.** The transcriptional binding sites of TEAD1 in GROα promoters.

**Additional file 3: Supplementary Figure S1.** MiR-141 is an exosomal miRNA derived from ovarian cancer cells. **Supplementary Figure S2.** MiR-141 induces stromal fibroblasts to have CAF-like properties. **Supplementary Figure S3.** GROα is the major proinflammatory chemokine in miR-141- expressing stroma. **Supplementary Figure S4.** YAP1 of Hippo signaling is the direct target of miR-141. **Supplementary Figure S5.** Conditional knockout (cKO) of Yap1 in stroma promotes tumor dissemination. **Supplementary Figure S6.** Depletion of CXCR1/2 impairs the oncogenic potential of OvCa cells induced by GROα.-enriched CM.

## Data Availability

The mass spectrometry proteomics dataset supporting the conlusions of this article is available in the ProteomeXchange Consortium via the PRIDE partner repository with the dataset identifier PXD026929. All other data supporting the conclusion of this article are including the uncropped Western Blot images are available in the Figshare website with the DOI 10.6084/m9.figshare.20963500or from the corresponding authors upon reasonable request.

## Abbreviations

αSMA: Alpha-smooth muscle actin
CULATR: Committee on the Use of Live Animals in Teaching and Research
CAFs: Cancer-associated fibroblasts
CXCR1/2: Human C-X-C Motif Chemokine Receptor 1/2
Cxcr1/2: Murine C-X-C Motif Chemokine Receptor 1/2
CM: Conditioned medium
DTT: Dithiothreitol
ELISA: Enzyme-Linked ImmunoSorbent Assay
EDTA: Ethylenediaminetetraacetic acid
EMMPRIN (CD147)/BSG: Extracellular matrix metalloproteinase inducer/basigin
ECM: Extracellular matrix
EVs: Extracellular vesicles
FAP: Fibroblasts activation protein
GAPDH: Glyceraldehyde 3-phosphate dehydrogenase
HGSOC: High-grade serous ovarian cancer
KO: Knockout
MET: Mesenchymal-to-epithelial transition
MMP-1/3/9: Matrix metalloproteinase-1/3/9
PBS: Phosphate buffered saline
PCR: Polymerase chain reaction
PVDF: Polyvinylidene difluoride
GROα/CXCL1: Growth-regulated oncogene-alpha/C-X-C motif chemokine ligand 1
OvCa: Ovarian cancer
OCM: Omental conditioned medium
SDS-PAGE: Sodium dodecyl sulfate polyacrylamide gel electrophoresis
SCCM: Stromal Cell Conditioned Media
TAZ: Tafazzin
TEA: Transcriptional Enhanced Associate
TEAD1: Transcriptional Enhanced Associate Domain 1
TME: Tumor microenvironment
YAP1: Yes-associated protein1
