# Supplementary material for: Tumor-secreted exosomal miR-141 activates tumor-stroma interactions and controls premetastatic niche formation in ovarian cancer metastasis
Source: Mol Cancer. 2023 Jan 9;22:4. doi: 10.1186/s12943-022-01703-9 (PMC9827705; doi:10.1186/s12943-022-01703-9)
Supplement: Supplementary file 1 — Additional file 1: Supplementary Table S1. Oligo sequences of plasmid cloning and qPCR. [file 12943_2022_1703_MOESM1_ESM.pdf]

Supplementary Table S1: Oligo sequences of plasmid cloning and qPCR.

Luciferase reporter plasmid construct sequences	
YAP1 Wild Type Sense(52bp)	CAATTCAGAATTCATACCAATCAGTGTTGAAACTCAAACATTGCAAAAGT
YAP1 Wild Type Antisense(58bp)	CTAGACTTTTGCAATGTTTGAGTTTCAACACTGATTGGTATGAATTCTGAATTGAGCT
YAP1 Mutant Type Sense(52bp)	CAATTCAGAATTCATACCAATCCCTCTTGAAACTCAAACATTGCAAAAGT
YAP1 Mutant Type Antisense(58bp)	CTAGACTTTTGCAATGTTTGAGTTTCAAGAGGGATTGGTATGAATTCTGAATTGAGCT
ChIP assay primers	
CBE1 Forward	GGGACACTGTATTTGGTCTCC
CBE1 Reverse	CCTTTCGGTTACTCAGGGGT
CBE2 Forward	GAGCTCTGAATCCCACTTGG
CBE2 Reverse	GCCGGATCCCACATAGAGTC
CBE3 Forward	GGGGCCCAATACTGAATACC
CBE3 Reverse	GCATAGCACATGACACCAGG
Luciferase reporter plasmid construced primers	
GROalpha CBE1-3 Forward	ACATGCTAGCACACCTTTCTAC
GROalpha CBE2-3 Forward	TAAAGCTAGCAGGTTAAATGAC
GROalpha CBE3 Forward	TTTTCGCTAGCAAGGGGGCCCAA
GROalpha CBEN Forward	ACTGACGCTAGCACTGGATTCAGC
GROalpha CBE Reverse	AGTGGAAGCTTGCGCTGAAGATACCA
CRISPR/Cas9 Knock-out sgRNA	
YAP1 Sense	CACCGGGACTCGGAGACCGACCTGG
YAP1Antisense	AAACCCAGGTCGGTCTCCGAGTCCC
TAZ Sense	CACCGGAGATGGCGTCTACCAGAAG
TAZ Antisense	AAACCTTCTGGTAGACGCCATCTCC
CRISPRi sgRNA	
Cxcr1 Sense1	CACCGACCCAGCTGGTGCCTCAGGT
Cxcr1 Antisense1	AAACACCTGAGGCACCAGCTGGGTC
Cxcr1 Sense2	CACCGGTACGCAACTCTGACTTCCT
Cxcr1 Antisense2	AAACAGGAAGTCAGAGTTGCGTACC
Cxcr1 Sense3	CACCGCTGGGTCCGTTCCTTGTGTG
Cxcr1 Antisense3	AAACCACACAAGGAACGGACCCAGC
Cxcr2 Sense1	CACCGGTCGTAGAACTACTGCAGGT
Cxcr2 Antisense1	AAACACCTGCAGTAGTTCTACGACC

Cxcr2 Sense2	CACCGATGCGTCATGCCGCTTCCCA
Cxcr2 Antisense2	AAACTGGGAAGCGGCATGACGCATC
Cxcr2 Sense3	CACCGGACGCTGTTTGTGAGCAGAG
Cxcr2 Antisense3	AAACCTCTGCTCACAAACAGCGTCC

TEAD1_TFBS_binding sites						
TEAD1_HUMAN.H10MO.D	CXCL1(-2k~+2k)	68	81	+	Forward: GGGACACTGTATTTGGTCTCC	
\					Reverse: CCTTTCGGTTACTCAGGGGT	from -1933 to -1920 AACATTCTAGCACA
TEAD1_HUMAN.H10MO.D	CXCL1(-2k~+2k)	861	874	+	Forward: GAGCTCTGAATCCCACTTGG	
					Reverse: GCCGGATCCCACATAGAGTC	from -1140 to -1127 GAAATTCCTGAGAG
TEAD1_HUMAN.H10MO.D	CXCL1(-2k~+2k)	989	1002	+	Forward: GGGGCCCAATACTGAATACC	
					Reverse: GCATAGCACATGACACCAGG	from -1012 to -999 TGCATGTCTGCTGC
