# Supplementary material for: Tumor-secreted exosomal miR-141 activates tumor-stroma interactions and controls premetastatic niche formation in ovarian cancer metastasis
Source: Mol Cancer. 2023 Jan 9;22:4. doi: 10.1186/s12943-022-01703-9 (PMC9827705; doi:10.1186/s12943-022-01703-9)
Supplement: Supplementary file 2 — Additional file 2: Supplementary Table S2. The transcriptional binding sites of TEAD1 in GROα promoters. [file 12943_2022_1703_MOESM2_ESM.pdf]

Supplementary Table S2: The transcriptional binding sites of TEAD1 in GROα promoters.

MOTIF TEAD1_HUMAN.H10MO.D																			
letter-probability matrix: alength= 4 w= 14 nsites= 30																			
0.05465587	0.463562753	0.204453	0.277328																
0.71659919	0.060728745	0.198381	0.024291																
0.206477733	0.623481781	0	0.17004																
0.906882591	0	0.093117	0																
0	0	0	1																
0.182186235	0	0.036437	0.781377																
0	0.720647773	0.068826	0.210526																
0.044534413	0.769230769	0.072874	0.11336																
0.457489879	0	0.048583	0.493927																
0.024291498	0.36437247	0.578947	0.032389																
0.344129555	0.396761134	0.080972	0.178138																
0.117408907	0.109311741	0.40081	0.37247																
0.032388664	0.477732794	0.279352	0.210526																
0.262145749	0.335020243	0.084008	0.318826																

URL http://hocomoco.autosome.ru/motif/TEAD1_HUMAN.H10MO.D												
#pattern name	sequence name	start	stop	strand	score	p-value	q-value	matched sequence	TSS	Genomic start	Genomic end	UCSC genome browser link
TEAD1_HUMAN.H10MO.D	CXCL1(-2k~+2k)	3664	3677	+	9.84783	0.000168	0.802	AACATTTCTCATGT	chr4:74,735,110	74736773	74736786	https://genome.ucsc.edu/cgi-bin/hgTracks?db=hg19&position=chr4:74736773-74736786
TEAD1_HUMAN.H10MO.D	CXCL1(-2k~+2k)	3450	3463	-	8.90217	0.000309	0.802	AACATTCTACCTTC	chr4:74,735,110	74736559	74736572	https://genome.ucsc.edu/cgi-bin/hgTracks?db=hg19&position=chr4:74736559-74736572
TEAD1_HUMAN.H10MO.D	CXCL1(-2k~+2k)	68	81	+	8.73913	0.000341	0.802	AACATTCTAGCACA	chr4:74,735,110	74733177	74733190	https://genome.ucsc.edu/cgi-bin/hgTracks?db=hg19&position=chr4:74733177-74733190
TEAD1_HUMAN.H10MO.D	CXCL1(-2k~+2k)	3490	3503	+	8.46739	0.000401	0.802	AATATTTCTGAGGA	chr4:74,735,110	74736599	74736612	https://genome.ucsc.edu/cgi-bin/hgTracks?db=hg19&position=chr4:74736599-74736612
TEAD1_HUMAN.H10MO.D	CXCL1(-2k~+2k)	2646	2659	-	8.3913	0.00042	0.802	CACATGCCAGTATT	chr4:74,735,110	74735755	74735768	https://genome.ucsc.edu/cgi-bin/hgTracks?db=hg19&position=chr4:74735755-74735768
TEAD1_HUMAN.H10MO.D	CXCL1(-2k~+2k)	861	874	+	8.26087	0.000452	0.802	GAAATTCCTGAGAG	chr4:74,735,110	74733970	74733983	https://genome.ucsc.edu/cgi-bin/hgTracks?db=hg19&position=chr4:74733970-74733983
TEAD1_HUMAN.H10MO.D	CXCL1(-2k~+2k)	989	1002	+	7.97826	0.000531	0.818	TGCATGTCTGCTGC	chr4:74,735,110	74734098	74734111	https://genome.ucsc.edu/cgi-bin/hgTracks?db=hg19&position=chr4:74734098-74734111
TEAD1_HUMAN.H10MO.D	CXCL1(-2k~+2k)	2519	2532	-	7.67391	0.000629	0.818	CCCATTCTTGAGTG	chr4:74,735,110	74735628	74735641	https://genome.ucsc.edu/cgi-bin/hgTracks?db=hg19&position=chr4:74735628-74735641
TEAD1_HUMAN.H10MO.D	CXCL1(-2k~+2k)	938	951	-	7.43478	0.000716	0.818	CACATTCCATTTAC	chr4:74,735,110	74734047	74734060	https://genome.ucsc.edu/cgi-bin/hgTracks?db=hg19&position=chr4:74734047-74734060

TEAD1_HUMAN.H10MO.D	CXCL1(-2k~+2k)	1908	1921	-	7.32609	0.000758	0.818	GGAGTTCCAGATCG	chr4:74,735,110	74735017	74735030	https://genome.ucsc.edu/cgi-bin/hgTracks?db=hg19&position=chr4:74735017-74735030
TEAD1_HUMAN.H10MO.D	CXCL1(-2k~+2k)	3688	3701	-	7.18478	0.000816	0.818	AACATTTTAGTTCT	chr4:74,735,110	74736797	74736810	https://genome.ucsc.edu/cgi-bin/hgTracks?db=hg19&position=chr4:74736797-74736810
TEAD1_HUMAN.H10MO.D	CXCL1(-2k~+2k)	1073	1086	-	7.17391	0.000821	0.818	ACCATTCCTGGTTC	chr4:74,735,110	74734182	74734195	https://genome.ucsc.edu/cgi-bin/hgTracks?db=hg19&position=chr4:74734182-74734195
TEAD1_HUMAN.H10MO.D	CXCL1(-2k~+2k)	1048	1061	-	6.79348	0.000997	0.935	TGCATAGCACATGA	chr4:74,735,110	74734157	74734170	https://genome.ucsc.edu/cgi-bin/hgTracks?db=hg19&position=chr4:74734157-74734170
