# Supplementary material for: Tumor-secreted exosomal miR-141 activates tumor-stroma interactions and controls premetastatic niche formation in ovarian cancer metastasis
Source: Mol Cancer. 2023 Jan 9;22:4. doi: 10.1186/s12943-022-01703-9 (PMC9827705; doi:10.1186/s12943-022-01703-9)
Supplement: Supplementary file 3 — Additional file 3: Supplementary Figure S1. MiR-141 is an exosomal miRNA derived from ovarian cancer cells. Supplementary Figure S2. MiR-141 induces stromal fibroblasts to have CAF-like properties. Supplementary Figure S3. GROα is the major proinflammatory chemokine in miR-141- expressing stroma. Supplementary Figure S4. YAP1 of Hippo signaling is the direct target of miR-141. Supplementary Figure S5. Conditional knockout (cKO) of Yap1 in stroma promotes tumor dissemination. Supplementary Figure S6. Depletion of CXCR1/2 impairs the oncogenic potential of OvCa cells induced by GROα.-enriched CM. [file 12943_2022_1703_MOESM3_ESM.pdf]

Supplementary Figures:

Tumor-secreted exosomal miR-141 activates tumor-stroma interactions and controls premetastatic niche formation in ovarian cancer metastasis

Yulan Mo[1], Leanne L Leung[1], Celia SL Mak[1], Xueyu, Wang[1], Wai-Sun Chan[1], Lynn MN Hui[1], Hermit WM Tang[1], Michelle KY Siu[1], Rakesh Sharma[2], Dakang Xu[3], Stephen KW Tsui[4], Hextan YS Ngan[1], Mingo MH Yung[1,*], Karen KL Chan[1,*] and David W Chan[1,4,5,*]

[1] Department of Obstetrics & Gynaecology, LKS Faculty of Medicine, The University of Hong Kong, Hong Kong SAR, China

[2] Centre for PanorOmic Sciences Proteomics and Metabolomics Core, Li Ka Shing Faculty of Medicine, The University of Hong Kong, Hong Kong SAR, China

[3] Faculty of Medical Laboratory Science, Ruijin Hospital, School of Medicine, Shanghai Jiao Tong University, Shanghai 200030, China

[4] School of Biomedical Sciences, The Chinese University of Hong Kong, Hong Kong SAR, China

[5] School of Medicine, The Chinese University of Hong Kong-Shenzhen, Shenzhen, 518172, China

*Correspondence: Dr. David W Chan, e-mail: dwchan@hku.hk, or dwchan@cuhk.edu.cn; and Dr. Karen KL Chan, e-mail: kklchan@hku.hk; and Dr. Mingo MH Yung, e-mail: h1094157@connect.hku.hk

A

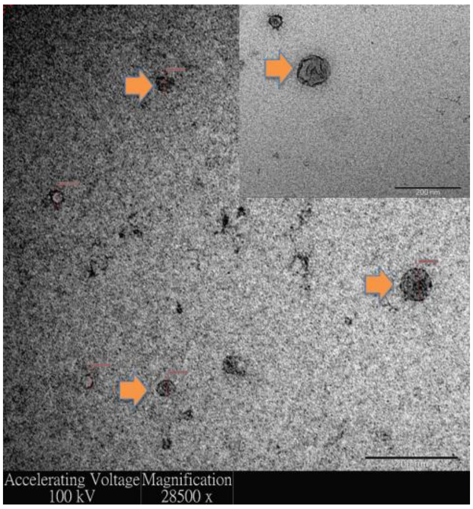
Accelerating Voltage
100 kV
Magnification
28500 x

B

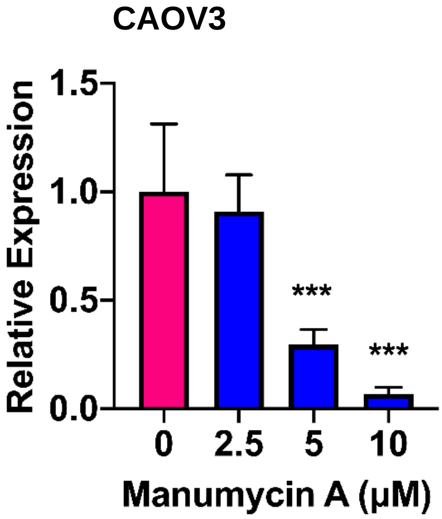
CAOV3
Relative Expression
1.5
1.0
0.5
0.0
***
***
0
2.5
5
10
Manumycin A (μM)

C

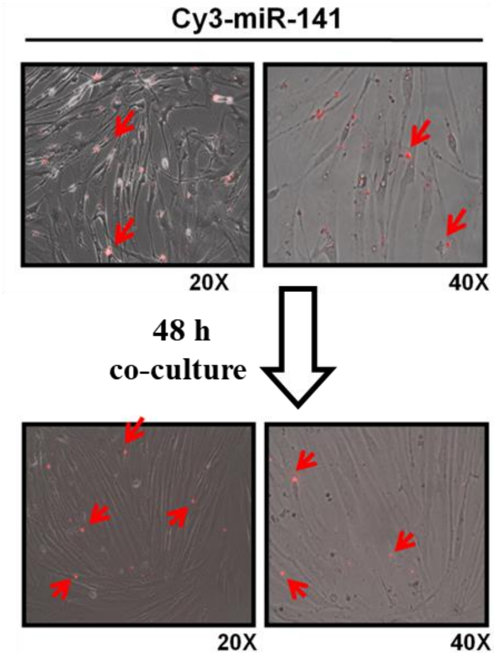
Cy3-miR-141
20X
40X
48 h
co-culture
20X
40X

Supplementary Figure S1. ***MiR-141 is an exosomal miRNA derived from ovarian cancer cells***

(A) Electron microscope images of exosomes found in the CM of ovarian cancer cells. Scale bar = 200nm.

(B) Co-treatment of Manumycin-A, a specific inhibitor of exosome biogenesis, in CAOV3 cells for 3 days showed a dose-dependent reduction of secreted miR-141 in the conditioned medium (CM) (n = 3, mean ± SEM, t-test, ***P<0.001).

(C) Fluorescent microscopies showed that Cy-3 labeled miR-141 was transfected to stromal fibroblast T HESCs and transferred between cells (Magnification 20x and 40x).

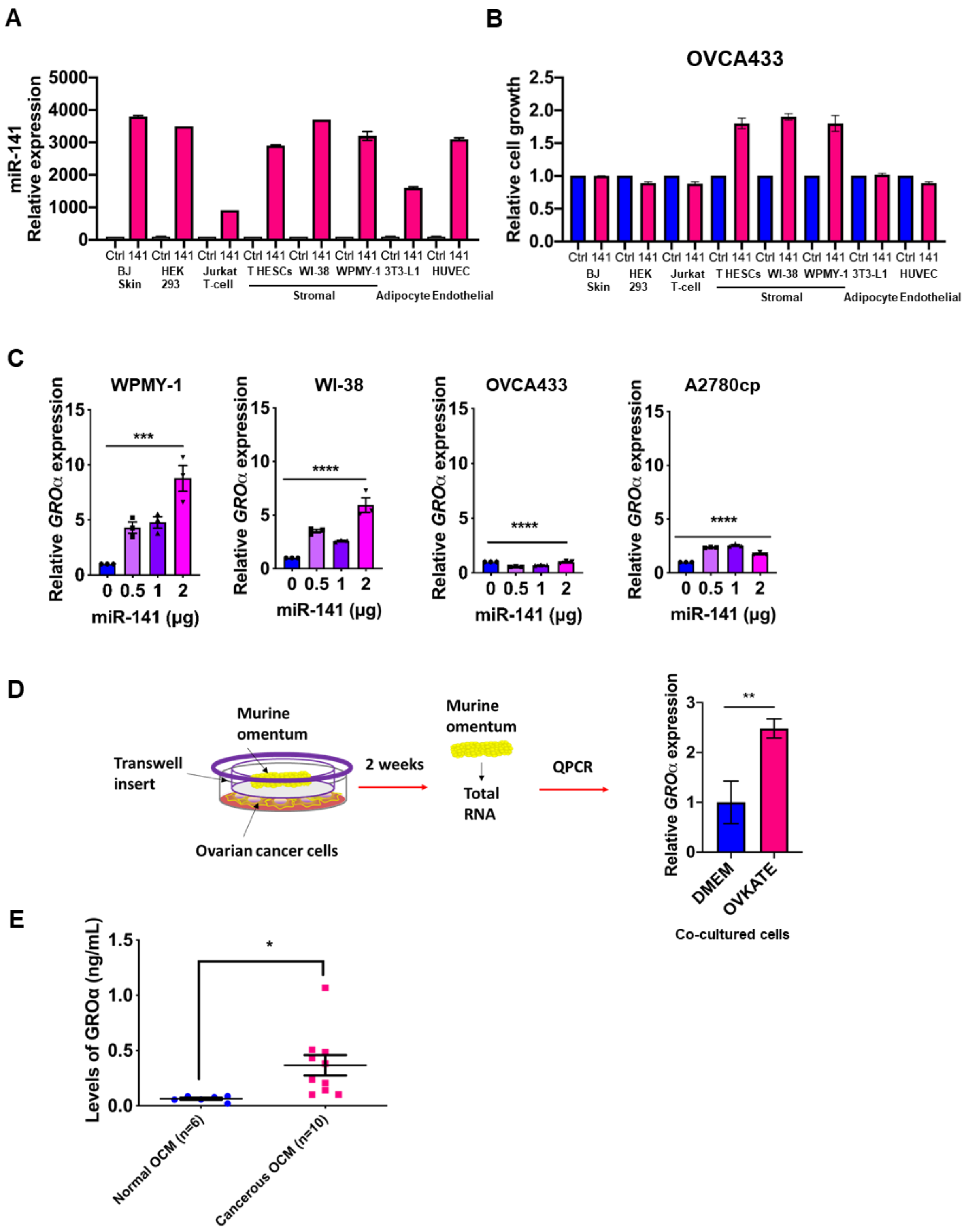
A
miR-141 Relative expression
5000
4000
3000
2000
1000
0
Ctrl 141 Ctrl 141 Ctrl 141 Ctrl 141 Ctrl 141 Ctrl 141 Ctrl 141 Ctrl 141
BJ Skin
HEK 293
Jurkat T-cell
T HESCs
WI-38
WPMY-1
3T3-L1
HUVEC
Stromal
Adipocyte
Endothelial
B
OVCA433
Relative cell growth
2.5
2.0
1.5
1.0
0.5
0.0
C
WPMY-1
WI-38
OVCA433
A2780cp
Relative GROα expression
miR-141 (μg)
0 0.5 1 2
***
****
D
Murine omentum
Transwell insert
Ovarian cancer cells
2 weeks
Total RNA
QPCR
**
DMEM
OVKATE
Co-cultured cells
E
Levels of GROα (ng/mL)
1.5
1.0
0.5
0.0
*
Normal OCM (n=6)
Cancerous OCM (n=10)

Supplementary Figure S2. ***MiR-141 induces stromal fibroblasts to have CAF-like properties***

(A) QPCR analysis of the transfection efficiency of pmR-ZsGreen1-miR-141 and pmR-ZsGreen1 (vector control) in a panel of non-cancer cell lines such as BJ, HEK293, Jurkat T-cell, T HESCs, WI-38, WPMY-1, 3T3-L1 and HUVEC cells (n = 3, mean ± SD).

(B) XTT cell proliferation assays showed the relative cell growth of OVCA433 cells upon a 5-day co-culture of CM from the above miR-141 overexpression cells and the respective vector control cells (n = 3, mean ± SD).

(C) QPCR analysis of the relative *GROα* expression in two human stromal cell lines (WPMY-1 and WI-38) and two ovarian cancer cell lines (OVCA433 and A2780cp) transfected with miR-141 overexpressing plasmid (0, 0.5, 1 and 2 μg) for 2 days (mean ± SEM, one-way ANOVA, *P<0.05, **P<0.01, ***P<0.001, ****P<0.0001).

(D) Schematic diagram and qPCR analysis indicated the workflow and the relative expression of *GROα* in the murine omentum co-cultured with OVKATE cells for two weeks in a Transwell insert (n = 3, mean ± SEM, t-test, **P<0.01).

(E) Graphic chart showed the relative expression of GROα in normal OCM (n = 6) and cancerous OCM (n = 10) by ELISA (mean ± SEM, t-test, *P<0.05).

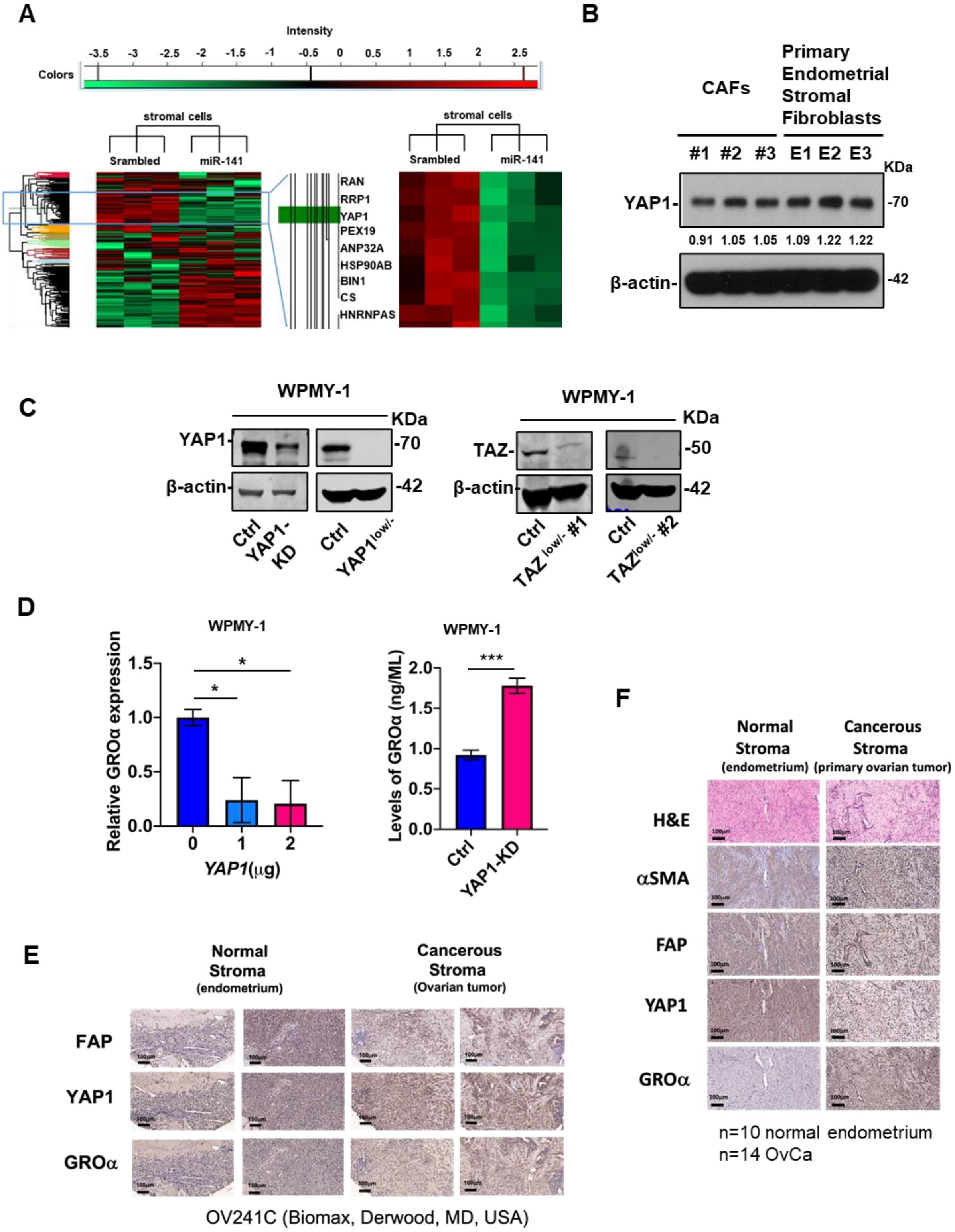
A
Intensity
-3.5 -3 -2.5 -2 -1.5 -1 -0.5 0 0.5 1 1.5 2 2.5
Colors
stromal cells
Srambled
miR-141
RAN
RRP1
YAP1
PEX19
ANP32A
HSP90AB
BIN1
CS
HNRNPAS
B
CAFs
Primary Endometrial Stromal Fibroblasts
#1 #2 #3 E1 E2 E3
KDa
YAP1-
-70
0.91 1.05 1.05 1.09 1.22 1.22
β-actin-
-42
C
WPMY-1
KDa
YAP1-
-70
β-actin-
-42
Ctrl
YAP1-KD
Ctrl
YAP1low/-
TAZ-
-50
Ctrl
TAZ low/- #1
Ctrl
TAZlow/- #2
D
WPMY-1
Relative GROα expression
YAP1(μg)
Levels of GROα (ng/ML)
Ctrl
YAP1-KD
E
Normal Stroma (endometrium)
Cancerous Stroma (Ovarian tumor)
FAP
YAP1
GROα
OV241C (Biomax, Derwood, MD, USA)
F
Normal Stroma (endometrium)
Cancerous Stroma (primary ovarian tumor)
H&E
αSMA
FAP
YAP1
GROα
n=10 normal endometrium
n=14 OvCa

Supplementary Figure S3. ***GROα is the major proinflammatory chemokine in miR-141-expressing stroma***

(A) Heatmap generated from LC-MS/MS proteomic analysis on WPMY-1 stromal fibroblasts with stable transfection of miR-141 showed YAP1 was a target of miR-141.

(B) Western blot analysis compared the expression of YAP1 between the CAFs extracted from the OvCa patients and the primary endometrial stromal fibroblasts. The Relative YAP1 expression (YAP1/β-actin) was quantified by ImageJ software.

(C) Western blot analysis showed the expression of YAP1 (left) and TAZ (right) in WPMY-1 cells with stable knockdown of YAP1 using shRNA (YAP1-KD), clones of CRISPR/Cas9-mediated gene knockout of YAP1 ($YAP1^{low/-}$), and TAZ ($TAZ^{low/-}$ #1 and $TAZ^{low/-}$ #2).

(D) Bar charts showed the qPCR analysis of the relative expression of *GROα* in WPMY-1 cells with transient transfection of YAP1 expressing plasmid (0 μg, 1 μg and 2 μg) (left) and the ELISA outcome on the relative expression of GROα in WPMY-1 cells with or without YAP1 stably knockdown (right) (n = 3, mean ± SEM, t-test, *P<0.05, ***P<0.001).

(E) The immunohistochemical analysis examined the relative expression of YAP1 and GROα between the normal stroma of endometrium and cancerous stroma of ovarian tumor in a commercial OvCa tissue microarray, OV241C (Biomax, Derwood, MD, USA). FAP was used as the stromal marker. Scale bar = 100 μm.

(F) Immunohistochemical analysis of the relative expression of YAP1 and GROα between the normal stroma of endometrium (n = 10) and cancerous stroma of primary ovarian tumor (n = 14). αSMA and FAP were used as stromal markers. Scale bar = 100 μm.

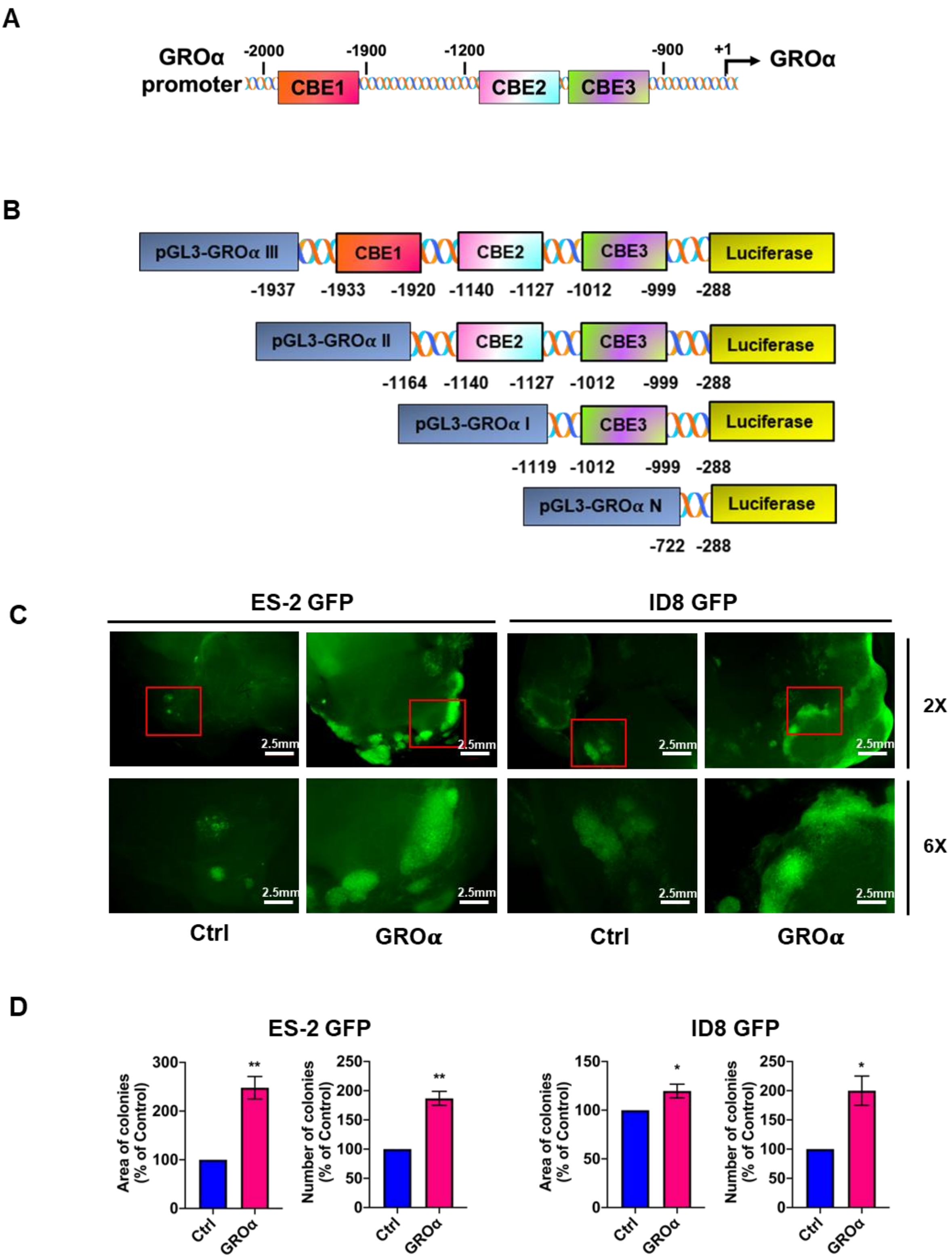
A
GROα promoter
-2000
-1900
-1200
-900
+1
CBE1
CBE2
CBE3
GROα
B
pGL3-GROα III
CBE1
CBE2
CBE3
Luciferase
-1937 -1933 -1920 -1140 -1127 -1012 -999 -288
pGL3-GROα II
CBE2
CBE3
Luciferase
-1164 -1140 -1127 -1012 -999 -288
pGL3-GROα I
CBE3
Luciferase
-1119 -1012 -999 -288
pGL3-GROα N
Luciferase
-722 -288
C
ES-2 GFP
ID8 GFP
2X
6X
2.5mm
Ctrl
GROα
Ctrl
GROα
D
ES-2 GFP
ID8 GFP
Area of colonies (% of Control)
Number of colonies (% of Control)
Ctrl
GROα
**
*

Supplementary Figure S4. ***YAP1 of Hippo signaling is the direct target of miR-141***

(A) Schematic diagrams of on GROα promoter which includes three predicted binding sites of TEAD (CBE1, CBE2, and CBE3).

(B) Schematic diagrams of the luciferase reporter plasmids containing the full-length and truncated GROα promoter.

(C) The fluorescent microscopy showed colony formation by GFP-labeled ES-2 and ID8 cells (ES-2 GFP and ID8 GFP) on the respective co-cultured murine omentum supplemented with or without human GROα recombinant protein (60 ng/mL). Scale bar = 2.5mm.

(D) The bar charts plotted the area and number of the colonies formed by ES-2 GFP and ID8 GFP on the respective co-cultured murine omentum supplemented with or without human GROα recombinant protein (60 ng/mL) (mean ± SEM, t-test, $*P<0.05$, $**P<0.01$).

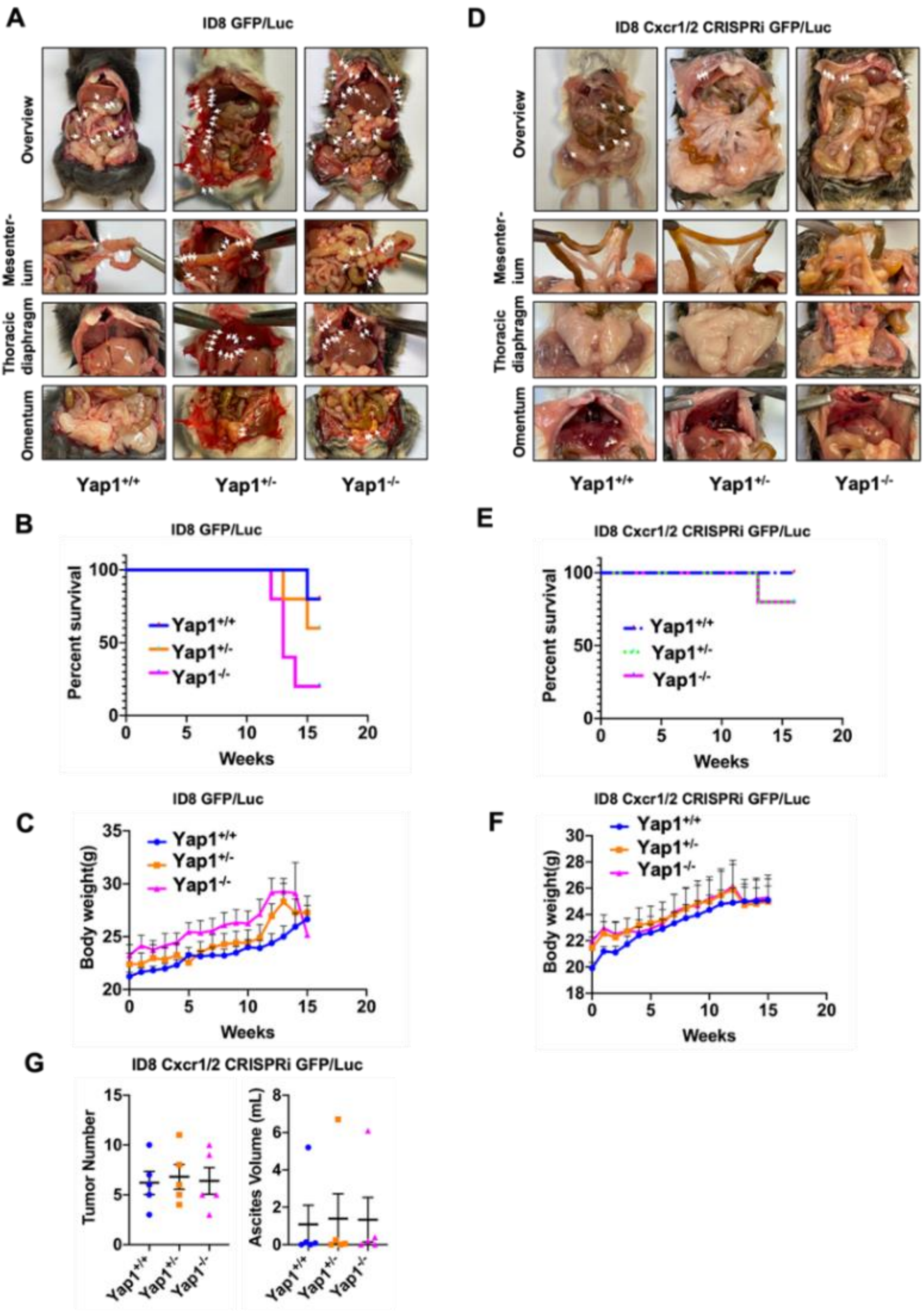
A
ID8 GFP/Luc
Overview
Mesenter-ium
Thoracic diaphragm
Omentum
Yap1+/+
Yap1+/-
Yap1-/-
D
ID8 Cxcr1/2 CRISPRi GFP/Luc
B
ID8 GFP/Luc
Percent survival
Weeks
E
ID8 Cxcr1/2 CRISPRi GFP/Luc
C
ID8 GFP/Luc
Body weight(g)
F
ID8 Cxcr1/2 CRISPRi GFP/Luc
G
ID8 Cxcr1/2 CRISPRi GFP/Luc
Tumor Number
Ascites Volume (mL)

Supplementary Figure S5. ***Conditional knockout (cKO) of Yap1 in stroma promotes tumor dissemination***

(A) The images displayed the tumor formation and dissemination in the stromal-specific Yap1 cKO mice (Yap1$^{+/-}$ and Yap1$^{-/-}$) and WT Yap1$^{+/+}$ mice upon intraperitoneal injection of GFP/Luciferase-labelled ID8 cells (ID8 GFP/Luc).

(B) Kaplan-Meier survival curves of overall survival analysis on Yap1$^{+/+}$, Yap1$^{+/-}$ and Yap1$^{-/-}$ mice after intraperitoneal injection with ID8 GFP/Luc cells (Yap1$^{+/+}$/Yap1$^{+/-}$: HR = 0.4310 (0.04456 ~ 4.170), P = 0.4593; Yap1$^{+/+}$/Yap1$^{-/-}$: HR = 0.1457 (0.02368 ~ 0.8970), P = 0.0330).

(C) Bodyweight of Yap1$^{+/+}$, Yap1$^{+/-}$ and Yap1$^{-/-}$ mice upon intraperitoneal injection of ID8 GFP/Luc cells.

(D) The images displayed the tumor formation and dissemination in the stromal-specific Yap1 cKO mice (Yap1$^{+/-}$ and Yap1$^{-/-}$) and WT Yap1$^{+/+}$ mice upon intraperitoneal injection of GFP/Luc-labelled ID8 Cxcr1/2 CRISPRi cells.

(E) Kaplan-Meier survival curves of overall survival analysis on Yap1$^{+/+}$, Yap1$^{+/-}$ and Yap1$^{-/-}$ mice after intraperitoneal injection with GFP/Luc-labelled ID8 Cxcr1/2 CRISPRi cells. (Yap1$^{+/+}$/Yap1$^{-/+}$: HR = 0 (-1.000 ~ -1.000), P = 0.3173; Yap1$^{+/+}$/Yap1$^{-/-}$: HR = 0.000 (-1.000 ~ -1.000), P = 0.3173).

(F) Bodyweight of Yap1$^{+/+}$, Yap1$^{+/-}$ and Yap1$^{-/-}$ mice upon intraperitoneal injection of GFP/Luc-labelled ID8 Cxcr1/2 CRISPRi cells.

(G) Dot plots showed the tumor number and ascites volume among Yap1$^{+/+}$, Yap1$^{+/-}$ and Yap1$^{-/-}$ mice after intraperitoneal injection with ID8 Cxcr1/2 CRISPRi GFP/Luc (n = 5, mean ± SEM).

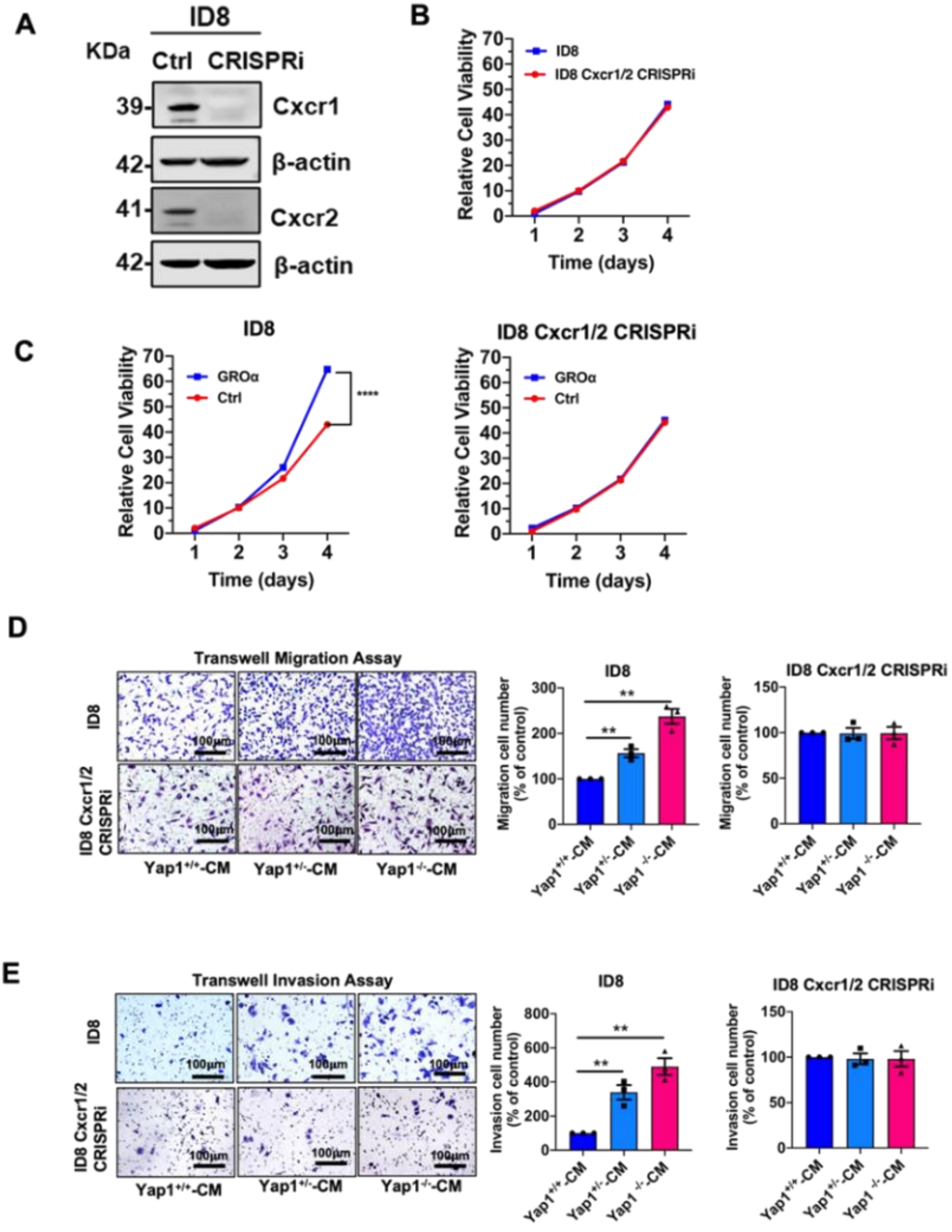
A
ID8
KDa
Ctrl
CRISPRi
39-
Cxcr1
42-
β-actin
41-
Cxcr2
42-
β-actin
B
ID8
ID8 Cxcr1/2 CRISPRi
Relative Cell Viability
Time (days)
C
ID8
GROα
Ctrl
****
Relative Cell Viability
Time (days)
ID8 Cxcr1/2 CRISPRi
GROα
Ctrl
Relative Cell Viability
Time (days)
D
Transwell Migration Assay
ID8
ID8 Cxcr1/2 CRISPRi
100μm
Yap1+/+-CM
Yap1+/--CM
Yap1-/--CM
ID8
Migration cell number (% of control)
**
**
ID8 Cxcr1/2 CRISPRi
Migration cell number (% of control)
E
Transwell Invasion Assay
ID8
ID8 Cxcr1/2 CRISPRi
100μm
Yap1+/+-CM
Yap1+/--CM
Yap1-/--CM
ID8
Invasion cell number (% of control)
**
**
ID8 Cxcr1/2 CRISPRi
Invasion cell number (% of control)

Supplementary Figure S6. ***Depletion of CXCR1/2 impairs the oncogenic potential of OvCa cells induced by GROα.*-enriched CM.**

(A) Western blot analysis compared the expression level of Cxcr1 and Cxcr2 in ID8 CRISPRi cells with ID8 parental cells (Ctrl).

(B) XTT cell proliferation assays indicated the relative cell growth of ID8 and ID8 Cxcr1/2 CRISPRi cells (n = 6, mean ± SEM, 2way ANOVA, **P*<0.05). N = 3 independent experiments.

(C) XTT cell proliferation assays indicated the relative cell growth of ID8 and ID8 Cxcr1/2 CRISPRi cells treated with recombinant GROα (n = 6, mean ± SEM, 2way ANOVA, *****P*<0.0001). N = 3 independent experiments.

(D) Transwell migration assay showed that the CM from the stromal-specific Yap1 cKO mice (Yap1$^{+/-}$-CM and Yap1$^{-/-}$-CM) significantly promoted higher cell migration capacities in ID8 parental cells than ID8 Cxcr1/2 CRISPRi cells as compared with their respective control using CM from WT Yap1$^{+/+}$ mice (n = 3, mean ± SEM, t-test, ***P*<0.01, ****P*<0.001). N = 3 independent experiments.

(E) Transwell invasion assay shows the mouse stromal CM from Yap1 cKO mice (Yap1$^{+/-}$-CM and Yap1$^{-/-}$-CM) promote more ID8 invasion capacities than ID8 Cxcr1/2 CRISPRi, Yap1$^{+/+}$ -CM was used as control) (n = 3, mean ± SEM, t-test, ***P*<0.01, ****P*<0.001). N = 3 independent experiments.
